# A case study of optimal design and techno-economic analysis of an islanded AC microgrid

**DOI:** 10.1038/s41598-025-94506-z

**Published:** 2025-04-11

**Authors:** M. S. Elborlsy, R. M. Mostafa, Mohamed A. Ghalib, Shimaa Barakat, H. E. Keshta

**Affiliations:** 1 Process Control Technology Department, Faculty of Technology and Education, Beni-Suef University, Beni-Suef, Egypt; 2 Electrical Engineering Department, Faculty of Engineering, Beni-Suef University, Beni-Suef, Egypt; 3 Electrical Engineering Department, Faculty of Engineering at Shoubra, Benha University, Banha, Egypt

**Keywords:** Renewable energy sources, Islanded AC microgrid, Diesel Generator (DG), Photovoltaic panel (PV), Microgrid (MG), Wind Turbine (WT), Fuel cell (FC), Electrical and electronic engineering, Energy grids and networks, Renewable energy

## Abstract

Microgrids (MGs) are essential in the distribution system by utilizing widely dispersed generation sources. Due to their economical and environmentally friendly attributes, Islanded AC MGs are commonly used to supply electricity to isolated locations independent of the primary grid. This study focuses on optimizing the configuration of an islanded AC MG to meet the electrical requirements of an international school in the New Administrative Capital, New Cairo, Egypt. Hybrid Optimization of Multiple Energy Resources (HOMER) software is employed to obtain the optimal size of the sources in the MG by minimizing the Levelized Cost of Energy (LCOE) and Total Net Present Cost (TNPC). According to the HOMER simulation results, a 200 kW PV system, a 180-kW wind turbine, a 50 kW FC, a 50 kW electrolyzer, a 50 kg hydrogen tank, a 180 kW DG, and a 686-kWh lead-acid battery form the optimal configuration of the islanded AC MG. The results reveal the contribution of each energy component to meeting the electricity demand, yielding an LCOE of $0.153/kWh and a TNPC of $1,775,300.00. The dynamic performance of the islanded microgrid is examined, introducing a Model Reference Adaptive Control based PI controller (MRAC-PI) to enhance transient response across all operational conditions. A comparative analysis is performed against traditional PI-PSO and PI-WOA controllers under load variations and changing weather conditions. The results indicate that the proposed control strategy effectively maintains system frequency and voltage amid various disturbances, improves dynamic performance, and achieves a balanced power generation and load demand. Additionally, the proposed controller demonstrates superior dynamic response, featuring reduced overshoot, undershoot, ITAE, and settling time compared to the others.

## Introduction

The escalating global population and the reliance on fossil fuels have raised serious ecological concerns, necessitating the search for sustainable and environmentally friendly energy sources. Renewable energy sources (RES), like PV and WT systems, have emerged as promising alternatives to nonrenewable fuels. However, these RES face challenges related to intermittent supply and the need for storage, particularly in arid regions^1^. MG systems offer a revolutionary energy production, delivery, and consumption solution. An MG comprises a set of distributed energy resources (DERs) and loads, functioning as a single, manageable unit. DERs encompass various micro-sources, including PV, WT, FC, DG, and battery energy storage system (BESS)^2^. As the world transitions toward renewable energy and grid resilience, MGs play a vital role by integrating RES into the electrical grid, reducing greenhouse gas emissions, and enhancing energy management^3^. Providing power to remote regions is crucial for strengthening the economy, creating jobs, reducing poverty, and improving living conditions. However, these areas often suffer from frequent disruptions, low quality, and power failures due to their distance from the conventional power grid. Expanding the grid to these regions is economically unviable. Implementing off-grid hybrid renewable energy systems (HRES) that combine PV, WT, and DG can address these power challenges and provide reliable and affordable electricity. Such independent HRES can also foster power utilization and economic development and overcome the limitations of limited fossil fuel reserves in rural and urban areas^4^. Designing an optimal HRES involves considering various factors, including identifying regions with the highest potential for renewable energy utilization, minimizing energy costs, and reducing emissions. Numerous tools, computer simulation software, and effective optimization methodologies have been employed to develop HRES models. Table 1 compares several simulation tools based on their characteristics and offerings^5^. One such software, HOMER, is widely used to evaluate the economic viability of HRES. HOMER enables the evaluation of financial feasibility and performance variations of HRES based on different energy sources^6^.

**Table 1:** Simulation programs for the design of HRES.

Software	Solar	Wind	Technical	Economic	Optimization	Free Trial
PV System	✔	x	✔	x	✔	✔
SolSim	✔	x	✔	✔	✔	x
Hybrid2	✔	✔	✔	x	x	✔
TRNsys	✔	✔	✔	x	x	✔
Hybrids	✔	✔	✔	x	✔	x
Hydrogels	✔	✔	✔	✔	✔	x
Ihoga	✔	✔	✔	✔	✔	x
Sam	✔	✔	✔	✔	x	✔
Rescreen	✔	✔	✔	✔	x	✔
Homer	✔	✔	✔	✔	✔	✔

The HOMER software is used to create the best system possible for remote areas based on the availability of RES. The best configuration for an isolated microgrid (IMG), including PV and BESS, was designed ^7–10^ to meet the energy demand in Saudi Arabia, India, Rwanda, and Algeria with high generation and low LCOE. In^11^, the WT/BESS hybrid system for the home in Xining, China, was shown to be the most optimal configuration for an IMG. In^12–16^, the PV/WT/ BESS hybrid configuration was identified as the most optimal solution for an IMG in Iran, India, India, Oman, and Pakistan. In^17^, the PV/BESS system was highlighted as optimal for residential and business loads and electric vehicle (EV) charging stations in Shah Alam, Malaysia. In^18–23^, the PV/ DG/ BESS hybrid system provided the most efficient configuration for IMGs in India, Nigeria, India, Benin, and the United Arab Emirates. In^24^, the WT/DG/ BESS hybrid system was shown to be optimal for urban residential loads in the United Kingdom. Meanwhile, the PV/WT/DG system was identified as the best configuration for rural community loads in Bhola, Bangladesh^25^. In^26^, the PV/DG/BESS hybrid system was optimal for remote community loads in Myanmar and Bangladesh. In^27–32^, demonstrated the effectiveness of the PV/WT/DG/BESS hybrid system across various regions, including Pakistan, Cameroon, Malaysia, Nigeria, Bangladesh, and India. In^33^, the PV/DG/BESS hybrid system was considered optimal for remote island community loads in Indonesia. In China, the PV/WT/DG/BESS hybrid system was recognized as the most efficient configuration for rural commercial, agricultural, community, and industrial loads^34^. In Saudi Arabia, the PV/FC/Hydrogen tank (HT)/ Electrolyzer (ELZ) hybrid system was shown to be optimal for residential applications^35^. Similarly, the PV/WT/FC/HT/ELZ hybrid system was the best solution for coastal residential loads in India^36^, and the PV/WT/FC/FC/BESS/HT/ELZ system was optimal for EV charging stations in India^37^. In^38^, the PV/WT/DG/BESS hybrid system was optimal for remote residential loads in Iran, while the PV/FC/HT/ELZ system was identified as ideal for domestic applications in Oman^39^. The PV/BESS system was recognized as the best configuration for remote commercial loads in Indonesia^40^. In Sub-Saharan Africa, the PV/WT/FC/BESS/HT/ELZ hybrid system was determined to be optimal for rural healthcare centers^41^. In^42^, the PV/WT/DG/BESS hybrid system was highlighted as the best configuration for remote residential loads in Algeria. The PV/FC/HT/ELZ system was noted as optimal for healthcare centers in Iran^43^. Lastly, studies In^44,45^ emphasized the optimal PV/DG/BESS hybrid system in India and Afghanistan. At the same time, in^46^ showed that the PV/WT/DG/BESS hybrid system was ideal for rural agricultural loads in Nigeria. The study focuses on Egypt’s RES potential and diverse climate. In^47^ examines eco-friendly hydrogen generation for electricity and hydrogen needs using RES in Egypt. Proposes sustainable freshwater production in Abuqir, Alexandria, using HRES Reverse Osmosis^48^. In^49^ compares renewable energy MG systems for New Sohag University, focusing on solar energy. In^50^ implements demand-side management for isolated systems in Egypt. The PV/WT/DG/BESS system has been identified as ideal for various applications, including residential loads in Aswan^51^, Hurghada^52^, and Western Sahara^53^, Egypt, as well as for industrial and residential loads in the Red Sea^54^ and residential communities in Marsa-Matruh^55^. Additionally, it is suitable for desalination plants in the Red Sea^56^. Meanwhile, the PV/WT/FC/HT/ELZ system is deemed optimal for Cairo International Airport^57^, and the WT/FC/HT/ELZ system is ideal for a remote small restaurant in Ras-Ghareb, Egypt^58^. Table 2 presents a comprehensive analysis of relevant research papers published in the last five years, which employed HOMER and MATLAB software to conduct stability and techno-economic analyses of isolated AC MGs.

**Table 2:** Comprehensive analysis of literature study.

Ref	Year	Method/Software	Objective(s)	Limitation(s)
^7^	2023	HOMER Pro & MATLAB	- Optimize RES based on location and implement a robust EMS to obtain MG stability	- Limited scope—Neglect of load variability—Neglect integration and reliability Issues—Simplistic models
^8^	2022	HOMER	- Presentation of an optimal off-grid HRES design and techno-economic analysis for rural loads	- Limited scope—Neglect integration and reliability Issues—Lack of comprehensive analysis—Simplistic models
^9^	2021	HOMER	- Designing an SPV plant for rural areas, providing the government with insights for similar projects	- Limited Scope—Neglect integration and reliability Issues—Lack of comprehensive analysis—Simplistic models
^10^	2020	HOMER & MATLAB	- Proposing a PSO-based DSM strategy to optimize an off-grid HRES for a residential building	- Limited scope—Simplistic models—Neglect integration and reliability Issues
^11^	2020	HOMER	- assessing the techno-economic feasibility of an off-grid WT-FC-BESS hybrid system	- High cost of energy—Neglect integration and reliability Issues Limited scope—Lack of comprehensive analysis
^12^	2023	HOMER	- Presentation of a comprehensive framework for an HRES integrating PV, WT, and biogas - Over 24 h, the suggested technique is thoroughly tested and programmed in an IMG	- High cost of energy—Limited case studies—Narrower focus—Lack of comprehensive analysis—Generalized findings—Neglect integration and reliability Issues—Poor results visualization—Lack of attention to user needs
^13^	2021	HOMER	- Examining challenges in HRES design and assessing the techno-economic feasibility for rural areas	- Lack of comprehensive analysis—Narrower focus—Neglect integration and reliability Issues
^14^	2020	HOMER	- Examining a Wave/PV/WT /BESS hybrid system for 3,000 households in three parts of Iran	- Lack of comprehensive analysis—Neglect integration and reliability Issues
^15^	2020	HOMER Pro	- Assessing an off-grid HRES for a reverse osmosis desalination plant	- Lack of comprehensive analysis—Narrower focus—Neglect integration and reliability Issues
^16^	2020	HOMER & MATLAB	- Presentation of an ideal design for an HRES to meet the home electricity demand of a residential area in Pakistan	- The MATLAB validation model’s component sizes differ from the HOMER’s optimal configuration, raising concerns about the validation process’s accuracy and reliability—Narrower focus
^17^	2023	HOMER Pro	- Using DSM to plan elastic loads and optimized MG design with an EV-inclusive load profile	- Limited scope—Lack of comprehensive analysis—Neglect integration and reliability Issues—Poor results visualization
^18^	2022	HOMER Pro	- Applying a probabilistic approach for sizing and assessing PV-based MGs, evaluating the environmental impacts of the configurations	- Neglect integration and reliability Issues—Narrower focus—Lack of comprehensive analysis—Poor results visualization
^19^	2021	HOMER	- Assessing the techno-economic viability of PV/WT/DG/ BESS hybrid systems for an IMG	- Lack of comprehensive analysis—Neglect integration and reliability Issues—Narrower focus
^20^	2021	HOMER	- Detailing the techno-economic feasibility and design of six HGIRES for rural electrification	- Lack of comprehensive analysis—Neglect integration and reliability Issues—Narrower focus
^21^	2020	HOMER	- Creating a conceptual framework, conducting a techno-economic analysis, and designing MG’s distribution network	- High cost of energy—Narrower focus—Lack of comprehensive analysis—Poor results visualization—Neglect integration and reliability Issues
^22^	2020	HOMER	- Analyzing the techno-economic feasibility of an HRES for rural electrification in Fouay village, Benin	- Lack of comprehensive analysis Neglect integration and reliability Issues—Narrower focus
^23^	2020	HOMER	- Optimizing and analyzing sensitivity for four PV, DG, and BESS systems, with and without tracking	- Neglect integration and reliability Issues—Narrower focus—Lack of comprehensive analysis
^24^	2020	HOMER	- Examining a hybrid power system that uses RES to supply a household’s electrical and heating needs	- High cost of energy—Neglect integration and reliability Issues—Narrower focus—Lack of comprehensive analysis
^25^	2022	TOPSIS with AHP	- Using TOPSIS with AHP to size an IMG for rural areas based on techno-economic parameters	- High cost of energy—Neglect integration and reliability Issues—Narrower focus—Lack of comprehensive analysis
^26^	2024	HOMER & MCDM	- Assessing hybrid energy systems for affordable, reliable power in four impoverished Asian nations	- Poor results visualization—Lack of comprehensive analysis—Neglect integration and reliability Issues
^27^	2024	HOMER Pro	- Examining three hybrid systems’ economic, technical, and emission performance	- Neglect integration and reliability Issues—Lack of comprehensive analysis
^28^	2023	HOMER & MATLAB	- Proposing a systematic optimization framework for an autonomous HRES in Dschang, Cameroon	- Neglect of Load Variability—Neglect integration and reliability Issues—The MATLAB validation model’s component sizes differ from HOMER’s optimal setup
^29^	2023	HOMER & MATLAB	- Using deterministic and stochastic optimization to determine the most cost-effective energy modules for meeting the load demand in Pulau Perhentian	- High Cost of Energy—Neglect integration and reliability Issues—The MATLAB validation model’s component sizes differ from the HOMER’s optimal configuration
^30^	2023	HOMER	- Modeling and optimizing a hybrid MG at the University in Nigeria for electricity generation	- Poor results visualization—Neglect integration and reliability Issues—Lack of comprehensive analysis
^31^	2023	HOMER Pro & ETAP	- Looking at different dispatch algorithms for an IMG, including DG, WT, BESS, and PV	- Lack of attention to user needs—Neglect integration and reliability Issues
^32^	2022	HOMER	- Creating the best possible HRES utilizing PV, WT, thermal loads, BESS, TLC, boiler, and DG	- Lack of comprehensive analysis—Neglect integration and reliability Issues
^33^	2022	HOMER Pro	- Feasibility of an HRES on a remote Indonesian island with existing DG and current diesel prices	- Lack of comprehensive analysis—Neglect integration and reliability Issues—Narrower focus
^34^	2020	HOMER	- Evaluating an off-grid HRES’s techno-economic viability for rural villages	- Lack of comprehensive analysis—Neglect integration and reliability Issues
^35^	2023	HOMER	- investigate the feasibility and cost-effectiveness of using FC with PV to power a load	- High cost of energy—Lack of comprehensive analysis—Neglect integration and reliability Issues
^36^	2021	HOMER	- Address hydrogen energy storage to solve power fluctuations using HOMER simulations	- Neglect integration and reliability Issues—Lack of comprehensive analysis
^37^	2022	HOMER	- Exploring hydrogen energy storage to manage power variability at an EV charging station in Delhi	- Lack of comprehensive analysis—Neglect integration and reliability Issues
^38^	2024	HOMER	- Evaluating off-grid HRES sustainability in remote Iran based on diesel prices and solar radiation	- Lack of attention to user needs—Lack of comprehensive analysis—Neglect integration and reliability Issues
^39^	2024	HOMER Pro & PVsyst	- Assessing the feasibility of combining floating PV with hydrogen storage for power generation	- High cost of energy—Lack of comprehensive analysis—Neglect integration and reliability Issues
^40^	2024	HOMER	- Using Baron Techno Park in Indonesia as a case study to build the ideal MG for remote 3 T locations	- Limited scope—Lack of comprehensive analysis—Neglect integration and reliability Issues
^41^	2024	HOMER	- Exploring HRES for electrifying rural healthcare in South Africa and Nigeria	- Lack of comprehensive analysis—Neglect integration and reliability Issues
^42^	2024	MATLAB (ISSA)	- Using the ISSA optimization technique to size an MG in Djelfa, Algeria	- Lack of comprehensive analysis—Neglect integration and reliability Issues
^43^	2024	HOMER	- Assessing RHCC layouts with a 10 kWh/day load in five climate-variable locations in Iran	- Lack of comprehensive analysis—Neglect integration and reliability Issues
^44^	2024	HOMER Pro	- PV efficiency will be assessed using performance metrics and a techno-economic analysis to design an IMG	- Narrower focus—Lack of comprehensive analysis—Neglect integration and reliability Issues
^45^	2024	HOMER Pro	- This study suggests a high-performing, reasonably priced MG for Afghanistan	- Narrower focus—Lack of comprehensive analysis—Neglect integration and reliability Issues
^46^	2024	HOMER	- Assesses HRES for Nigeria’s agriculture, addressing rural electricity issues	- Lack of comprehensive analysis—Neglect integration and reliability Issues
^47^	2023	HOMER Pro	- Produce green hydrogen for isolated hydrogen and electrical loads using RES	- Narrower focus—Lack of comprehensive analysis—Neglect integration and reliability Issues
^48^	2023	HOMER Pro	- Machine learning is applied to enhance load forecasting and adapt to increasing water demand	- Neglect integration and reliability Issues—Poor results—Lack of comprehensive analysis—Narrower focus
^49^	2019	HOMER	- Comparing two renewable energy MG systems for New Sohag University focusing on PV generation	- Poor results visualization—Lack of comprehensive analysis—Neglect integration and reliability Issues
^50^	2024	HOMER Pro	- Examining the viability and design of HRES for rural electrification in an Egyptian village	- Poor results visualization—Lack of comprehensive analysis—Neglect integration and reliability Issues
^51^	2024	HOMER Pro	- Presentation of an optimal design for IMGs in a remote Nubian village in Aswan, Egypt	- Lack of comprehensive analysis—Neglect integration and reliability Issues
^52^	2021	HOMER	- The techno-economic studies combining DG, WT, and SPVS for Hurghada City, Egypt	- Poor results visualization—Lack of comprehensive analysis—Neglect integration and reliability Issues
^53^	2019	HOMER	- The ideal hybrid MG size for rural electrification in Abu-Monqar, Egypt, is presented in this study	- Poor results visualization—Lack of comprehensive analysis—Neglect integration and reliability Issues
^54^	2022	HOMER	- Designing and assessing the feasibility of eight HRES using HOMER software	- Poor results visualization—Lack of comprehensive analysis—Neglect integration and reliability Issues
^55^	2020	HOMER Pro &MATLAB	- Offering a clear, four-phase, scientific framework for designing HRES in an Egyptian community	- The system’s scope is restricted by lacking technologies like FC, limiting efficiency and versatility
^56^	2020	HOMER Pro	- Evaluating off-grid power systems and optimizing HRES for a reverse osmosis facility	- Lack of comprehensive analysis—Neglect integration and reliability Issues
^57^	2024	HOMER Pro	- Developing a deterministic method to optimize HRES size, focusing on 1-year simulations	- Lack of comprehensive analysis—Neglect integration and reliability Issues
^58^	2023	HOMER	- Evaluating green hydrogen as a possible fuel and energy storage medium for Egypt’s RES	- Lack of comprehensive analysis—Neglect integration and reliability Issues

The literature review highlighted several research gaps and limitations in these specific points, as described here:

- Lack of comprehensive analysis: focusing solely on technical issues without incorporating economic and environmental analysis or concentrating only on financial considerations without thoroughly examining or validating the suggested models.
- Limited Case Studies: concentrates on speculative scenarios or does not include case studies from the real world.
- Limited Scope: looking at one technology (PV, wind, or DG) without considering a hybrid strategy.
- Narrower focus: ignore the possible advantages of hybrid configurations with cutting-edge parts like PV-WT-DG-BESS -FC-ELZ-HT, which can restrict the usefulness and efficacy of the proposed solutions.
- Generalized Findings: Other researchers’ results are generic and not adapted to use cases or geographical areas.
- Neglect of Load Variability: did not take generation and load variations into sufficient account.
- Neglect integration and reliability Issues: The operational and technological difficulties of integrating diverse energy sources are not included, and the reliability and quality of the power supply are not discussed in detail.
- Simplistic Models: employ too basic models that fail to represent the intricacy of actual energy systems adequately.
- Poor Results Visualization: The findings are presented less understandably, devoid of illustrative charts or explicit representations.
- Lack of Attention to User Needs: Its conclusions do not match the requirements or inclinations of end users.
- high cost of energy: This high price may put off potential customers or investors, particularly in areas with limited resources, diminishing the solution’s overall viability and appeal.

Considering all the above, the contributions and scope of the work that is being presented are summed up as follows:

- Comprehensive analysis: The paper integrates technological and economic factors, offering a holistic approach that addresses the limitations of focusing solely on one aspect. HOMER is employed for optimal component sizing, thus contributing to the development of economically viable MG solutions. Subsequently, MATLAB uses the component sizes determined by HOMER to model the operational and technological challenges of integrating multiple energy sources.
- Advanced modeling techniques: The study employs sophisticated modeling techniques that capture the complexity of real-world systems, utilizing both HOMER for economic analysis and MATLAB/Simulink for technical analysis.
- Real-World Case Study: The study analyzes the specific electrical requirements of an international school in New Cairo and provides a concrete application of the proposed MG configuration.
- proposing a non-linear adaptive controller, MRAC-PI controller, to enhance the transient response of frequency over

a wide range of operating points. To demonstrate the efficiency of the proposed controller, its performance is examined, evaluated, and compared with the PI-PSO and PI-WOA controllers.

- The location’s significance is due to its representation of emerging urban development’s prioritizing sustainable energy solutions, making it relevant for similar settings worldwide.
- Hybrid technology integration: Including advanced components such as FC and ELZ alongside PV, WT, DG, and BESS allows for a more versatile and efficient energy system.
- Consideration of load variability: The research thoroughly accounts for generation and load variations, enhancing the proposed system’s reliability and robustness.
- Focus on reliability and addressing Integration Issues: The paper discusses the reliability and quality of power supply in detail, addressing a common gap in recent literature. It also explores the operational and technological challenges of integrating multiple energy sources using MATLAB software, providing valuable insights for practical implementation.
- User-centric approach: the research aligns its conclusions with the specific needs and preferences of an international school in New Cairo, ensuring that the proposed solutions are practical and user-friendly.
- Cost-effective solutions: The study contributes to developing economically viable MG solutions by aiming to minimize the LCOE and TNPC.

This paper’s remaining parts are organized as follows. A thorough explanation of the technique and a description of the suggested power system are provided in Sect. “Optimal sizing and economic analysis using HOMER PRO”. This includes the site details, evaluation of energy resources, load profile, and the economic and mathematical models used for the suggested system’s elements. Section “Feasibility study” presents the results and discussions from the HOMER software and the optimization achievements. Financial analysis is also included in this section, along with the rated capacity in Kilowatt (kW) and quantity of units for each type of system component, as well as the expenses associated with the ideal system, such as TNPC and LCOE. Section “Mathematical modeling” focuses on the MATLAB system under study, specifically, the islanded AC MG system configuration and the load frequency control mechanism, and Sect. “Optimization and economic model” presents MATLAB simulation results that demonstrate how the load frequency control maintains constant AC bus voltage and frequency, achieves active power balancing among the components of an islanded AC MG, and ensures adequate AC voltage quality in the presence of load and generation disturbances. Finally, Sect. “Model constraints” concludes this work.

## Optimal sizing and economic analysis using HOMER PRO

**Fig. 1:**
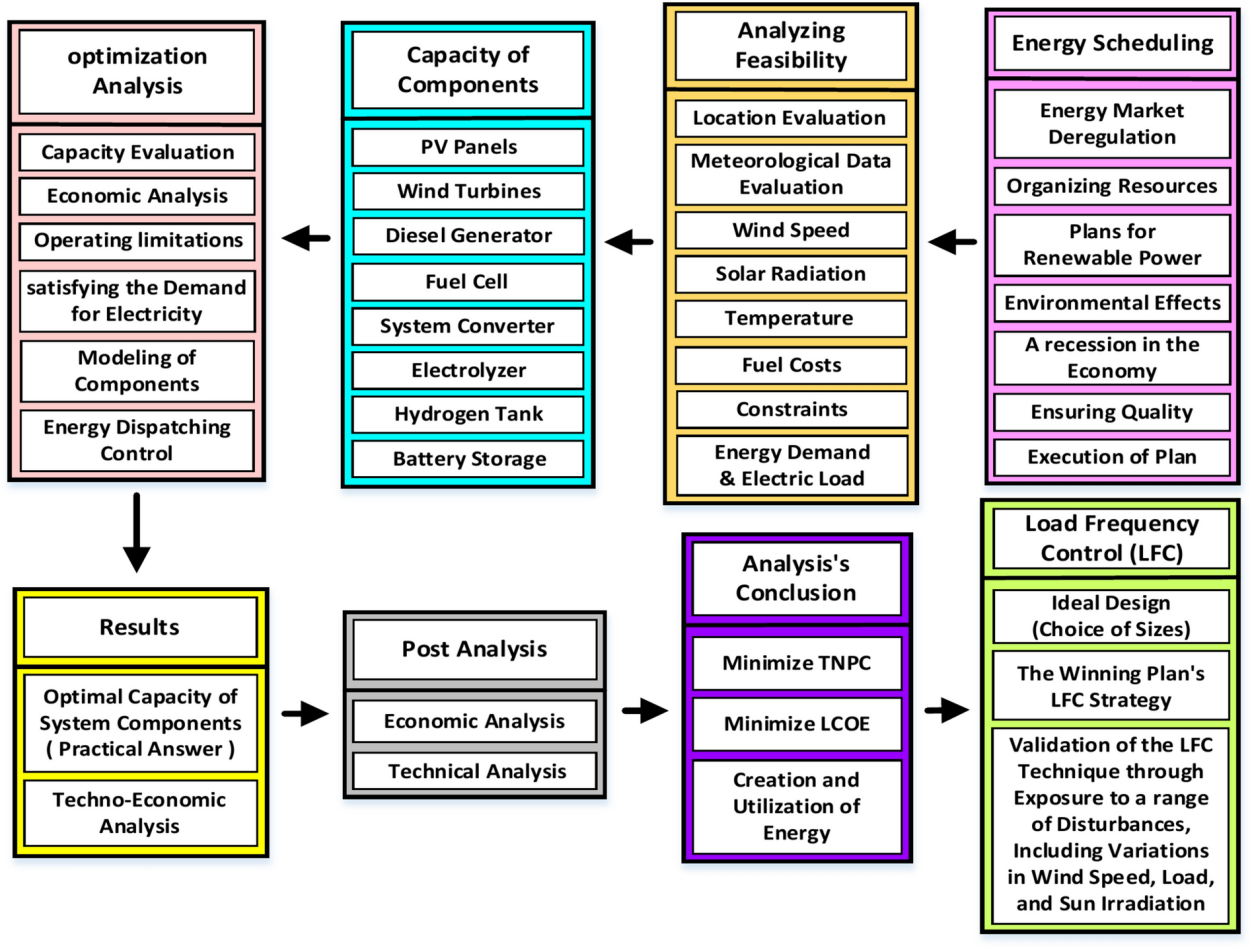
The suggested system for managing, controlling, and optimizing islanded AC MG.

Figure 1 outlines the recommended approach for optimizing component size and load frequency control (LFC) in an off-grid AC MG. The process involves evaluating real-time weather data and the load profile of the proposed location. Optimization goals, constraints, and feasible HRES configurations are determined, and a detailed model of multiple components is developed. Based on technical and economic analysis, the most practical model is selected. LFC is then implemented for the chosen configuration plan. Finally, the suggested control approach is validated by subjecting it to various disturbances, such as changes in solar irradiance, load fluctuations, and wind speed variations. The HRES consists of PV, WT, and FC as the primary energy sources, with a DG and BESS used to supply power during PV and wind fluctuations. Power converters are vital in transferring energy between DC and AC buses.

### Feasibility study

This section provides a framework for assessing the country’s power policy, describing the site, and analyzing the load requirements that must be met.

### Site description

**Fig. 2:**
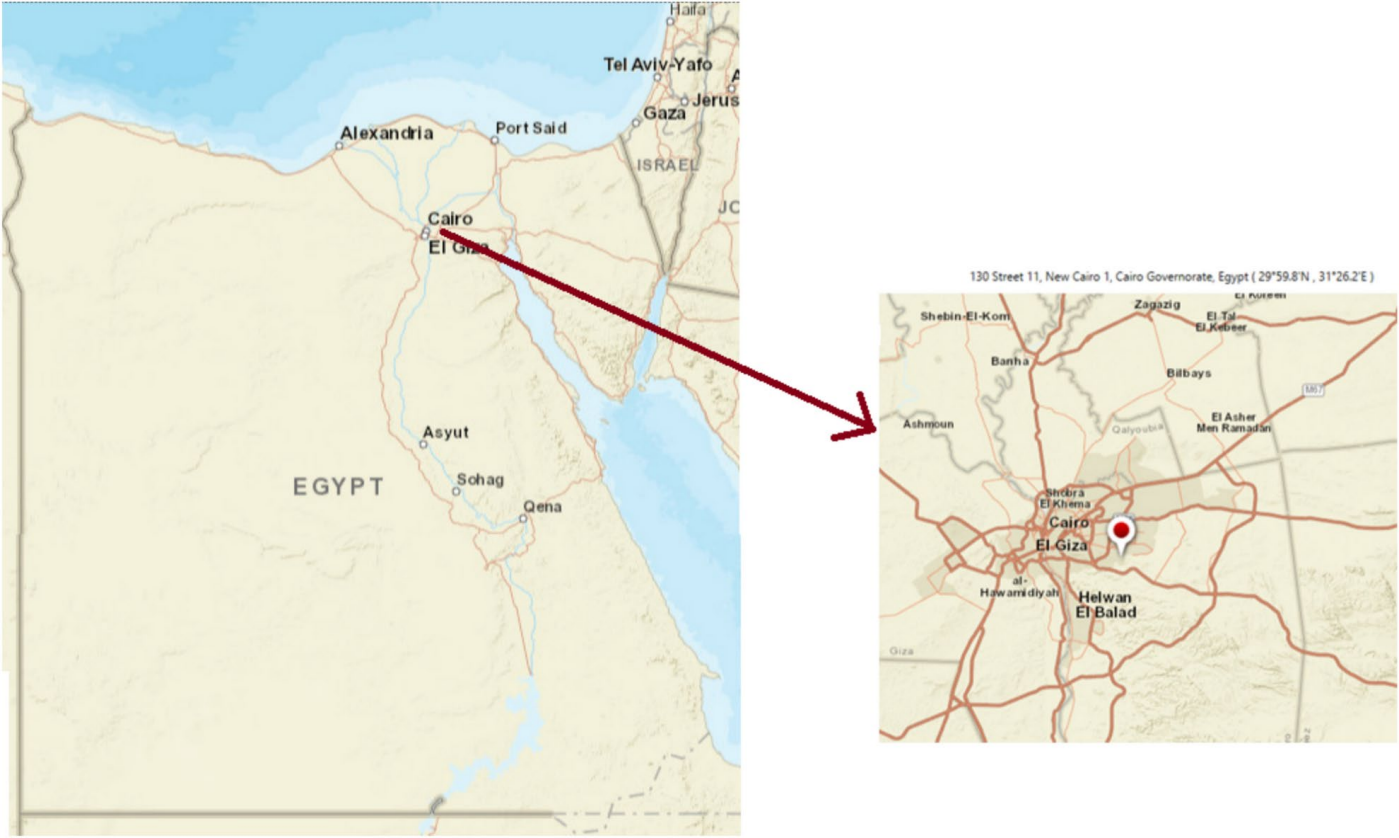
Location of the case study site. This map was generated using HOMER Pro × 64 3.14.5 software (https://homerenergy.com/).

The suggested system under investigation was based on a case study of an international school, 130 Street 11, New Cairo 1, Cairo Governorate, Egypt. As shown in Fig. 2, the case study’s precise coordinates are 29°59.8’N and 31°26.2’E. This location was selected because it offers ideal conditions for dependable year-round access to PV and wind resources.

### Solar energy data

**Fig. 3:**
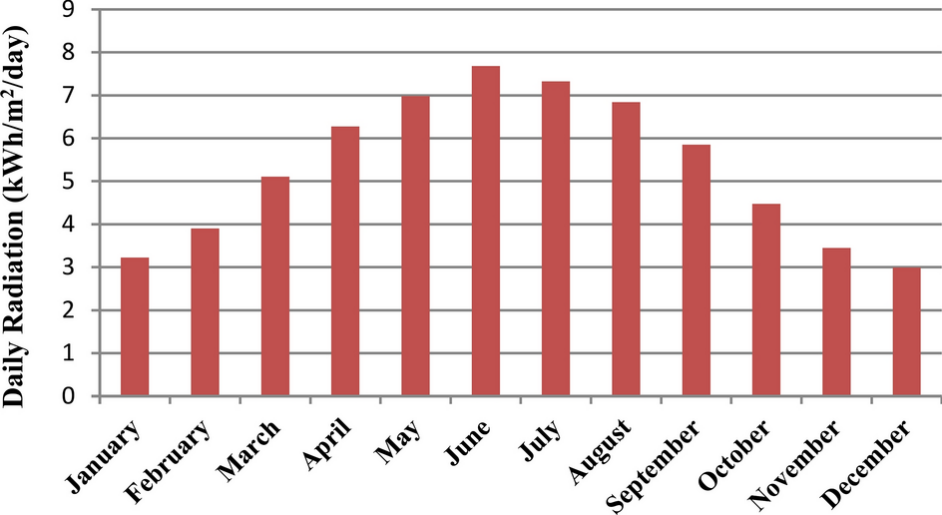
Monthly average solar radiation of the suggested location.

Solar resource data was obtained from the National Aeronautics and Space Administration (NASA) Surface Meteorology and Solar Energy website^59^. This source is widely recognized for its reliability and comprehensiveness in providing solar irradiance data. The scaled data of the worldwide solar radiation at the chosen point is displayed in Fig. 3. Data analysis reveals that June experiences the highest solar radiation, averaging (7.68 kWh/m^2^/day), which is crucial for maximizing solar energy production during the summer months. In contrast, December shows the lowest solar radiation levels at (2.996 kWh/m^2^/day), reflecting the seasonal variations in solar availability. Overall, the site receives a yearly average solar radiation of (5.342 kWh/m^2^/day). This average is significant, as it indicates a favorable environment for solar energy generation, making the location suitable for implementing solar technologies.

### Wind energy data

**Fig. 4:**
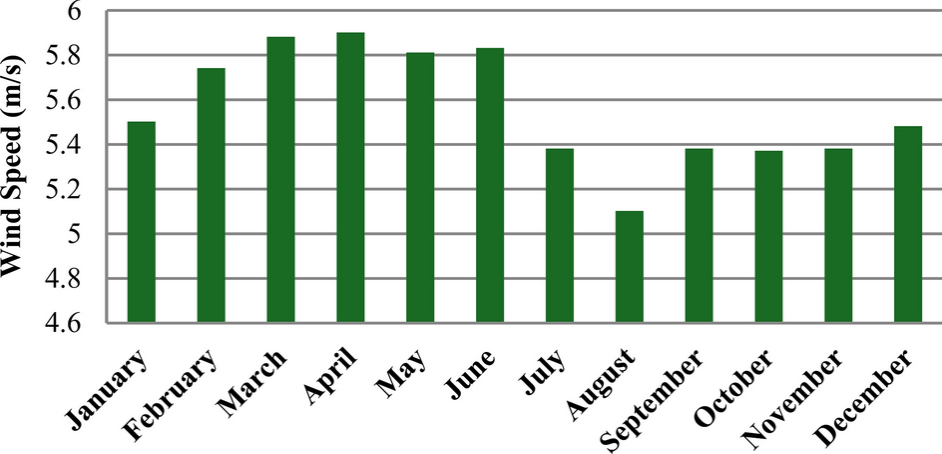
Average monthly wind speed of the suggested location.

The Cairo Governorate exhibits significant potential for wind resources, with a mean hourly wind speed of 5.56 m/s. This wind speed indicates a favorable environment for wind energy generation, making it an attractive site for developing wind power projects. Figure 4 illustrates the monthly mean wind speeds at the suggested location, providing a detailed overview of the wind resource variability throughout the year. It is evident from the graph that the highest average hourly wind speed occurs in April at 5.91 m/s; this peak suggests that spring months may provide optimal conditions for wind energy generation. Meanwhile, the wind speed was recorded at its lowest in August at 5.1 m/s. This seasonal variation highlights the region’s dynamic nature of wind resources, which various meteorological factors can influence. The figure also emphasizes a complementary relationship between wind speeds and solar radiation. Wind speeds are relatively high during months when solar radiation is typically low, such as in the summer. This synergy between wind and solar resources is advantageous for meeting load demand throughout the year. By capitalizing on both energy sources, a more reliable and balanced energy supply can be achieved, ultimately enhancing the overall efficiency of the energy system. This complementary nature supports the optimization of energy generation. It contributes to reducing reliance on conventional fossil fuels, paving the way for a more sustainable energy future in the Cairo Governorate. The ability to harness both wind and solar resources effectively positions the region as a promising candidate for renewable energy initiatives that can meet the growing energy demands of its population.

### Load profile

**Fig. 5:**
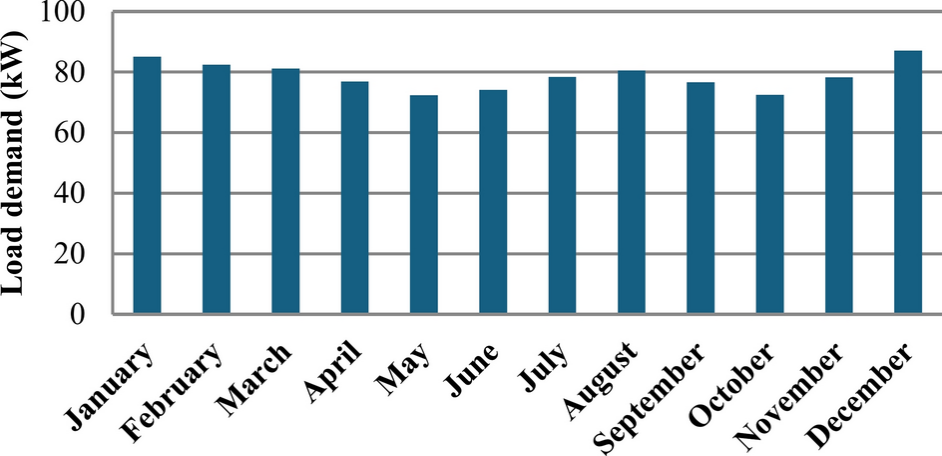
The selected site’s monthly average load.

To accurately represent the energy consumption patterns in the research area, the average daily load demand is utilized to construct an hourly load profile for the entirety of the year. This comprehensive approach allows for a granular understanding of energy usage, reflecting fluctuations that occur throughout different times of the day and various seasons. The research area demonstrates an average hourly load of around 78.8 kWh, indicating a consistent demand for energy throughout the day with a peak load reaching 162.13 kW. Figure 5 presents the monthly average load profile for the selected site, based on estimated energy consumption for an international school in the New Administrative Capital, New Cairo, Egypt. This load profile is representative of actual consumption patterns, further strengthening our analysis. By analyzing the load profile in conjunction with seasonal demand variations, this study provides valuable insights into the energy needs of the area. Understanding these patterns is essential for designing an efficient energy system that can effectively meet the load demand, optimize resource allocation, and ensure reliability in energy supply throughout the year. This analysis also lays the groundwork for integrating RES, as it highlights the times when energy generation must be maximized to align with consumer demand.

## Mathematical modeling

This section provides the economic and mathematical models of the suggested system’s elements. The system’s LCOE was computed using the discounted cash flow analysis approach, considering the project’s lifespan and constituent parts. A 25-year project lifespan has been taken into consideration. To improve the accuracy of the estimates, economic data such as the inflation rate (${I}_{f}$) and interest rate (${I}_{r}$) are also considered.

### Solar generation system

PV panel output power can be estimated using a variety of models. Instead, we use a straightforward model that only needs two inputs: solar radiation and the surrounding temperature. To calculate the power of the PV panels, use the following equation^19^.

1 $${P}_{PV}={P}_{STC}{f}_{PV} \left(\frac{G}{{G}_{STC}}\right) \left[1+K\left(T-{T}_{STC}\right)\right]$$

${P}_{PV}$ do PV panels generate the power, ${P}_{STC}$ is the rated power of PV panels at Standard Test Condition (STC), ${f}_{PV}$ represents the PV derating factor (0.8), G is the actual solar irradiance received by PV panels (kW/m^2^), ${G}_{STC}$ is the solar irradiance at STC (1 kW/m^2^), ${T}_{STC}$ is the cell temperature at reference conditions (25 °C), and K is the temperature coefficient. The cell temperature (T) is calculated using the equation presented in^24^ involving the ambient temperature (${T}_{amp}$).

2 $$T={T}_{amp}+ \left[\frac{NOCT-20}{0.8} G\right]$$

The PV generator’s annual power production ( $${P}_{PVannual} )$$ can be represented as follows^60^.

3 $${P}_{PVannual}= {\sum }_{t=1}^{T}\left({N}_{PV}\times {f}_{PV}\times {P}_{PVr}\right)\left(\frac{G}{{G}_{STC}}\right)\left(1+K\left(\left({T}_{amp}+ G\times \left(\frac{NOCT-20}{800}\right)*1000\right)-{T}_{STC}\right)\right),T=8760$$

The rated module output (${P}_{PVr}$) in kW, the number of PV modules ${(N}_{PV})$, the symbols represent normal operating cell temperature (NOCT) in degrees Celsius (℃).

The net present cost ($${NPC}_{PV}$$) of the PV system is determined by adding the initial capital cost ($${IC}_{PV}$$) and the operation and maintenance costs ($${O\&MC}_{PV}$$), the initial price of the PV system ($${IPR}_{PV}$$), and $${( OMP}_{PV})$$ is the operation and maintenance price of the PV system ($). Replacement costs are nonexistent because the PV system’s lifespan is the same as the projects. The $${NPC}_{PV}$$ can be calculated using the following formula:

4 $${NPC}_{PV}={O\&MC}_{PV}+{IC}_{PV}$$

5 $${NPC}_{PV}={N}_{PV}\times \left[{IPR}_{PV}+\sum_{n=0}^{25}\frac{{OMP}_{PV}}{{\left(1+ \frac{{I}_{r}-{I}_{f}}{1+{I}_{f}}\right)}^{n-1}}\right]$$

### Wind generation system

The WT harnesses wind energy to generate electricity, utilizing rotating rotor blades and a generator. It plays a vital role in the MG by capturing and using renewable energy to power the entire system. The formula determines the power produced by the WT^14^.

6 $${P}_{WT}= \left\{\begin{array}{c}0 {v}_{ci}>v\\ {\frac{{P}_{r \times \left({v}^{3}-{{v}_{cutin}}^{3}\right)}}{{{v}_{rated}}^{3}-{{v}_{cutin}}^{3}} v}_{rated}\ge v\ge {v}_{couout}\\ {{P}_{r} v}_{rated}\le v\le {v}_{couout}\\ 0 {v}_{couout}<0v\end{array}\right.$$

where the output power of the WT (kW), the WT’s rated power (kW), the WT generator’s local speed, rated wind speed, cut-out wind speed, and cut-in wind speed are respectively described by the variables ($${P}_{WT}, {P}_{r},$$ v, $${v}_{rated}$$, $${v}_{couout}$$, and $${v}_{cutin})$$.

Given that WT has a 15-year lifespan, it must be replaced every 15 years. It is possible to compute the WT system’s net present cost $\left({NPC}_{WT} \right)$ as follows:

7 $${NPC}_{WT}={O\&MC}_{WT}+{IC}_{WT}+ {RC}_{WT}$$

8 $${NPC}_{WT}={N}_{WT}\times \left[{IPR}_{WT}+\sum_{n=0}^{25}\frac{{OMP}_{WT}}{{\left(1+ \frac{{I}_{r}-{I}_{f}}{1+{I}_{f}}\right)}^{n-1}}+\frac{{RP}_{WT}}{{\left(1+ \frac{{I}_{r}-{I}_{f}}{1+{I}_{f}}\right)}^{15}}\right]$$

where the initial capital cost of WT is ($${\text{IC}}_{\text{WT}}$$), the initial price of the WT system ($${IPR}_{WT}$$), $${( OMP}_{WT})$$ is the operation and maintenance price of the WT system ($), The number of wind turbines $${(N}_{WT})$$, The replacement costs of the WT ( $${RC}_{WT})$$, Replacement price of WT ($) is $$( {RP}_{WT})$$,and the maintenance cost of WT is ($${O\&MC}_{WT}$$).

### The batteries bank system modeling

Batteries, which are very energy-efficient and straightforward to set up, are the only storage agents used in this study. When energy is scarce, extra power from RES is utilized to start charging the battery. The discharge and charge settings of a battery can be ascertained using Equations. (9) and (10), correspondingly^10,14^:

9 $${E}_{b}\left(t+1\right)= {E}_{b}\left(t\right)\times \left(1-\sigma \right)- \left[\frac{{E}_{l}\left(t\right)}{{\eta }_{cnv}}-{E}_{g}\left(t\right)\right]{\times \eta }_{BD}$$

10 $${E}_{b}\left(t+1\right)= {E}_{b}\left(t\right)\times \left(1-\sigma \right)+ \left[{E}_{g}\left(t\right)-\frac{{E}_{l}\left(t\right)}{{\eta }_{cnv}}\right]{\times \eta }_{BC}$$

where ${E}_{l}\left(t\right)$ and ${E}_{g}\left(t\right)$ stand for generated power and energy demand, respectively, ${E}_{b}\left(t\right)$ is the state of charge (SOC) of a battery at each time step t, The battery’s discharge and charge efficiencies are represented by ${\eta }_{BD}$ and ${\eta }_{BC}$. The battery’s self-discharge σ, and the converter’s efficiency ${\eta }_{cnv}$. The battery bank has no replacement costs because its 25-year lifespan equals the project’s lifetime. The battery bank’s net present cost ${( NPC}_{BAT })$ is computed as follows:

11 $${NPC}_{BAT}={O\&MC}_{BAT}+{IC}_{BAT}$$

12 $${NPC}_{BAT}={N}_{BAT}\times \left[{IPR}_{BAT}+\sum_{n=0}^{25}\frac{{OMP}_{BAT}}{{\left(1+ \frac{{I}_{r}-{I}_{f}}{1+{I}_{f}}\right)}^{n-1}}\right]$$

where the initial capital cost of BESS is ($${\text{IC}}_{\text{BAT}}$$), the initial price of the BESS ($${IPR}_{BAT}$$), $${( OMP}_{BAT})$$ is the operation and maintenance price of BESS ($), The number of battery banks $${(N}_{BAT})$$,and the operating and maintenance cost of BESS is ($${O\&MC}_{BAT}$$).

### Diesel Generation system

The DG serves as the MG’s backup power source, supplying electricity when generated power from other resources is low, demand is high, and BESS is depleted. The power output equation for the DG used in HOMER is displayed in^30^:

13 $${P}_{DG}={C}_{O\&M}+ \frac{{C}_{r}}{{R}_{L}}+ {F}_{n}{X}_{gen}{C}_{eff}$$

where $${C}_{eff}$$ is the efficient fuel cost ($/L), $${R}_{L}$$ is the generator lifespan (hrs), $${C}_{r}$$ is the replacement cost ($), $${X}_{gen}$$ is the generator capacity (kW), $${F}_{n}$$ is the intercept fuel curve coefficient (fuel/hr/kW), and $${C}_{O\&M}$$ is the maintenance and operating cost ($/hr).

### Inverter and converters

Converters enable bidirectional transfer between AC and DC power. Inverter/converters facilitate energy flow between DC and AC buses. Power converters are necessary when both DC and AC components are present. System modeling considers anticipated loads, efficiency, and capacity of the inverter. The inverter ${P}_{inv}$ capacity is established by^30^.

14 $${P}_{inv}=\frac{{E}_{L}\left(max\right) }{{\eta }_{DC/AC}}$$

where the load system’s maximum energy need (kWh), and the converter’s efficiency are represented by ${E}_{L}\left(max\right)$, and ${\eta }_{DC/AC}$ respectively.

### Electrolyzer model

The electrochemical converter splits water into oxygen and hydrogen. Daytime electrolysis with purified water and RES generates stored hydrogen. A polymer electrolyte membrane (PEM) electrolysis extracts hydrogen from water and solar power, compressing and storing it. DC voltage splits water, combining protons and electrons for hydrogen production. The system utilizes porous anodes, polymer film, flow fields, and plates for water transport^35,36^. The following formula can be used to get the ELZ net present cost (${NPC}_{ELZ}$):

15 $${NPC}_{ELZ}={O\&MC}_{ELZ}+{IC}_{ELZ}+ {RC}_{ELZ}- {S}_{ELZ}$$

16 $${NPC}_{ELZ}={N}_{ELZ}\times \left[{IPR}_{ELZ}+\sum_{n=0}^{25}\frac{{OMP}_{ELZ}}{{DF}^{n-1}} +\frac{{RP}_{ELZ}}{{DF}^{{T}_{ELZ}}}-{S}_{ELZ}\right]$$

17 $${S}_{ELZ}={RP}_{ELZ}\left(\frac{{T}_{ELZ}-\left({T}_{proj}-{T}_{ELZ}\times INT\left(\frac{{T}_{proj}}{{T}_{ELZ}}\right)\right)}{{T}_{ELZ}}\right)$$

where the initial capital cost of the ELZ is ($${\text{IC}}_{\text{ELZ}}$$), the initial price of the ELZ ($${IPR}_{ELZ}$$), $${( OMP}_{ELZ})$$ is the operation and maintenance price of ELZ ($), The number of ELZ $${(N}_{ELZ})$$, The replacement costs of the ELZ ( $${RC}_{ELZ})$$, The replacement price of the ELZ ($) is $$\left({RP}_{ELZ}\right)$$, The ELZ’s salvage value ($${S}_{ELZ}$$), the discount factor (DF),$${T}_{proj}$$ = 25.

years,$\text{ELZ operational life }\left({T}_{ELZ}\right)$,$\text{ELZ operational life }\left({T}_{ELZ}\right),$ and the operating and maintenance cost of the ELZ is (${O\&MC}_{ELZ}$).

### Fuel cell generation system

FC converts chemical energy to electrical energy. Oxidizers and fuel produce fuel and water. ELZ separate cathodes for circuit completion. Pure sewage. Hydrogen interaction transports electrons. Protons are generated in the anode. Water is released by hydrogen, oxygen, and electron combination^36,37^. PEM/FCs are chosen for efficiency, lifespan, size, and weight. They are used in backup power and transmission networks^61^.

18 $$\text{FC operational life }({T}_{FC})=\frac{ FC life time}{FC hours throughout a year}$$

It’s feasible to Determine the net present cost of the FC system ${( NPC}_{FC}$ ) and the fuel cell’s salvage value (${S}_{FC}$) as follows:

19 $${NPC}_{FC}={O\&MC}_{FC}+{IC}_{FC}+ {RC}_{FC}- {S}_{FC}$$

20 $${NPC}_{FC}={N}_{FC}\times \left[{IPR}_{FC}+\sum_{n=0}^{25}\frac{{OMP}_{FC}}{{DF}^{n-1}} + \frac{{RP}_{FC}}{{DF}^{{T}_{FC}}}-{S}_{FC}\right]$$

21 $${S}_{FC}={RP}_{FC}\left(\frac{{T}_{FC}-\left({T}_{proj}-{T}_{FC}\times INT\left(\frac{{T}_{proj}}{{T}_{FC}}\right)\right)}{{T}_{FC}}\right)$$

where the initial capital cost of FC is ($${\text{IC}}_{\text{FC}}$$), the initial price of the FC is ($${IPR}_{FC}$$), $${( OMP}_{FC})$$ is the operation and maintenance price of FC ($), The number of FC’s $${(N}_{FC})$$, The replacement costs of the FC ( $${RC}_{FC})$$, Replacement price of FC ($) is $$\left({RP}_{FC}\right)$$, and the operating and maintenance cost of FC is ($${O\&MC}_{FC}$$).

### Hydrogen storage tank (HT)

Large-scale fixed hydrogen storage is indispensable for fulfilling hydrogen’s potential as a worldwide energy transporter. An approximate lifespan of 25 years is projected for the hydrogen container. The tank’s dimensions are determined through simulations that aim to meet the optimal design criteria. The following equations outline the operational principles of the HT^58^:

22 $${Q}_{{H}_{2}}\left(t\right)={m}_{{H}_{2}}\left(t\right)-{f}_{{H}_{2}}\left(t\right)-{L}_{{H}_{2}}\left(t\right)$$

23 $${T}_{level}\left(t\right)={Q}_{{H}_{2}}\left(t\right)$$

24 $${T}_{level,new}\left(t\right)=\sum_{t=1}^{t=(t-1)}{T}_{level}\left(t\right)+{T}_{level}\left(t\right)$$

where, ${Q}_{{H}_{2}}\left(t\right)$ presents the net mass flow rate of hydrogen entering or respond the ELZ and leaving the tank for the FC. ${m}_{{H}_{2}}\left(t\right)$ or respond to the ELZ’s output mass flow rate at a specific time. ${f}_{{H}_{2}}\left(t\right)$ signifies the input mass flow rate to the hydrogen FC simultaneously, measured in kg/h. ${L}_{{H}_{2}}\left(t\right)$ denotes the rate of hydrogen consumption in kg/h. ${T}_{level}\left(t\right)$ presents the amount of hydrogen added to the tank during a given time interval within one hour while ${T}_{level,new}\left(t\right)$ signifies the updated tank level at the current time step. The first part of Eq. (24) indicates the remaining hydrogen kept in the tank before this computation hour, while the second part of the equation represents the final hydrogen held during the present hour.

### Optimization and economic model

**Fig. 6:**
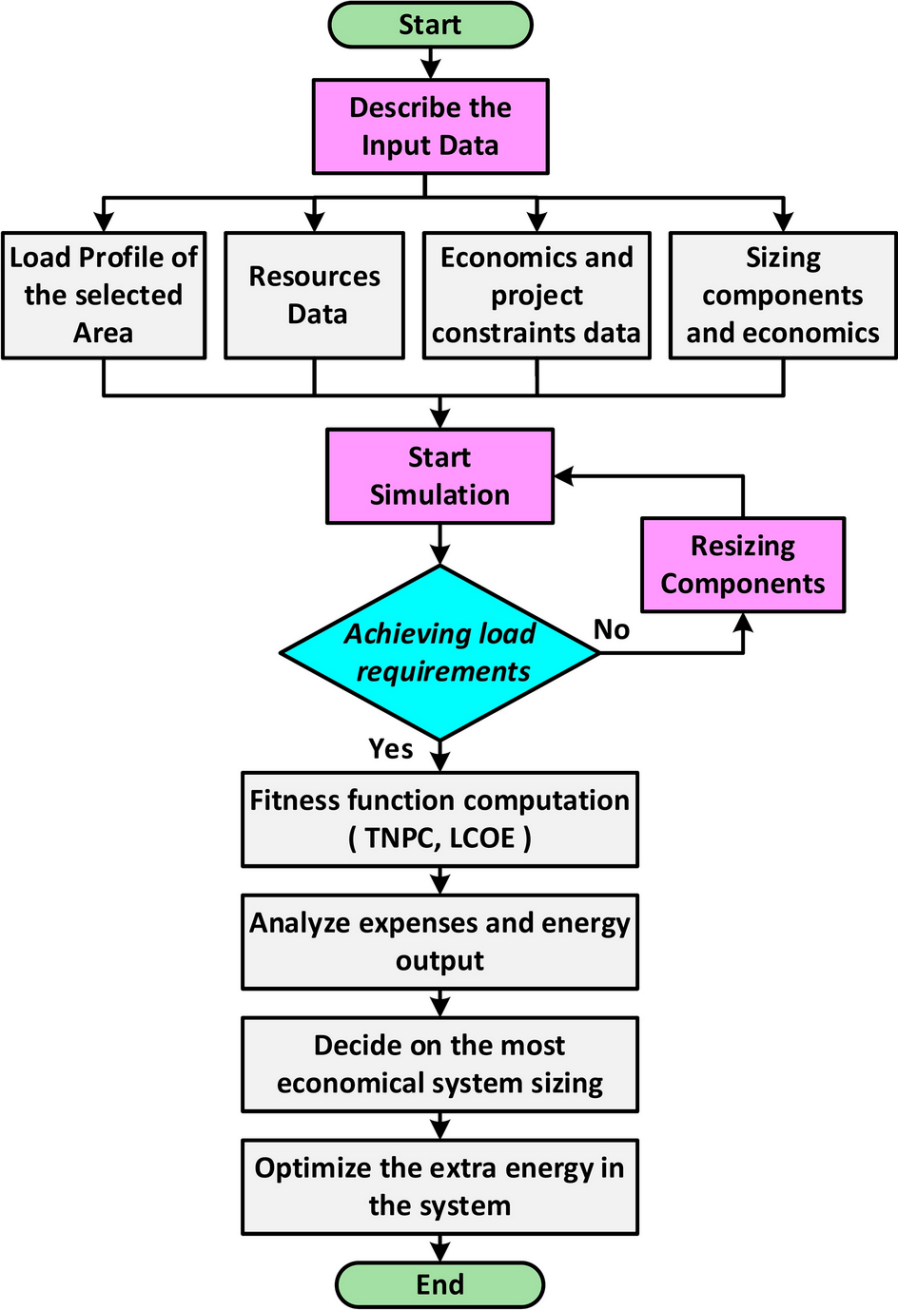
Flowchart of the HOMER algorithm.

The optimization process determines the best system configuration that meets intended load limitations by simulating various configurations in HOMER-Pro, as presented in Fig. 6. Unrealistic solutions are eliminated, and feasible configurations are ranked based on the lowest LCOE and TNPC. The objectives are to determine Proper values for user-controlled decision-making variables, such as DG size, PV module, FC, system converter, ELZ, HT, WT number, battery number, and dispatch strategy. HOMER-Pro analyzes these variables to identify the most suitable configuration for the required demand. The program defines LCOE as the mean cost per kWh of usable electrical power generated by the system. The program calculates the LCOE by dividing the yearly expense of power production by the total amount of helpful power produced. The following formula is used to get the LCOE and TNPC^10,11^.

25 $$LCOE=\frac{ {C}_{Anualized}}{ {E}_{Served}}$$

26 $${C}_{Anualized}=TNPC\times CRF$$

27 $$TNPC=\frac{{C}_{Anualized}}{CRF}$$

where, ${C}_{Anualized}$ is the total annualized cost, CRF is the capital recovery factor, and ${E}_{Served}$ is the annual energy served. The following equations^25^ provide the capital recovery factor (*CRF*) and the annual interest rate (*r*).

28 $$CRF=\frac{ r{\left(1+r\right)}^{N}}{ {\left(1+r\right)}^{N}-1}$$

29 $$r=\frac{ {r}{\prime}-f}{ 1+f}$$

where (*N*) is the project lifespan in years, $(r{\prime})$ is the rate of nominal interest expressed as a percentage, and ($f$) is the annual inflation rate expressed as a percentage. The project’s duration in this study is 25 years, with a 21.25% interest rate and a 35.12% inflation rate per the Central Bank of Egypt^62,63^.

## Model constraints

To ensure a consistent balance of power throughout each hour, all electricity requirements must be satisfied by utilizing appropriate sources of power such as PV, WT, FC, and DG, as described in this paper. The equation provided below describes the principle behind achieving this objective.

30 $${P}_{Load}= \sum {P}_{DG}+{P}_{WT}+{P}_{PV}+{P}_{FC }\pm {P}_{BESS}$$

The operation of the battery is limited by two conditions: (i) the SOC must adhere to safe limits to prevent overcharging or over-discharging, as depicted in the equations provided in Eq. (31), and (ii) the amount of energy stored per hour is bound by specific constraints outlined in Equations. (32), (33) and (34)^30,55^.

31 $${SOC}_{min}\le SOC\left(t\right) \le {SOC}_{max}$$

32 $${E}_{bat.min}\le {E}_{bat}\left(t\right) \le {E}_{bat.max}$$

33 $${E}_{bat.min}= {E}_{bat.max} \left(1-DOD\right)$$

34 $${E}_{bat.cap}= {E}_{bat.max}$$

In the equations, $({E}_{bat})$ represents battery capacity (kWh), $({E}_{bat.min})$ is the minimum permissible battery capacity, ${(E}_{bat.max})$ is the maximum permissible battery capacity, $({SOC}_{min})$ is the minimum SOC, ${(SOC}_{max})$ is the maximum SOC, and (DOD) is the depth of discharge.

### HOMER results and discussions

HOMER Pro models and optimizes MG systems, considering both financially and technically feasible Integration of RES and risk reduction. It assesses the components, fuel costs, system structure, demand characteristics, and environmental variables in a single run. The simulation offers insightful data on cost reductions, risk mitigation, and system performance. The investigation’s conclusions are compiled in this part of the paper, which begins with the optimization achievements and then on to the economic analysis. These sections list each component’s rated capacity (kW) and the quantity of units for each system component. These component tables provide the expenses of the ideal system, including the TNPC and LCOE.

### Optimization results

**Fig. 7:**
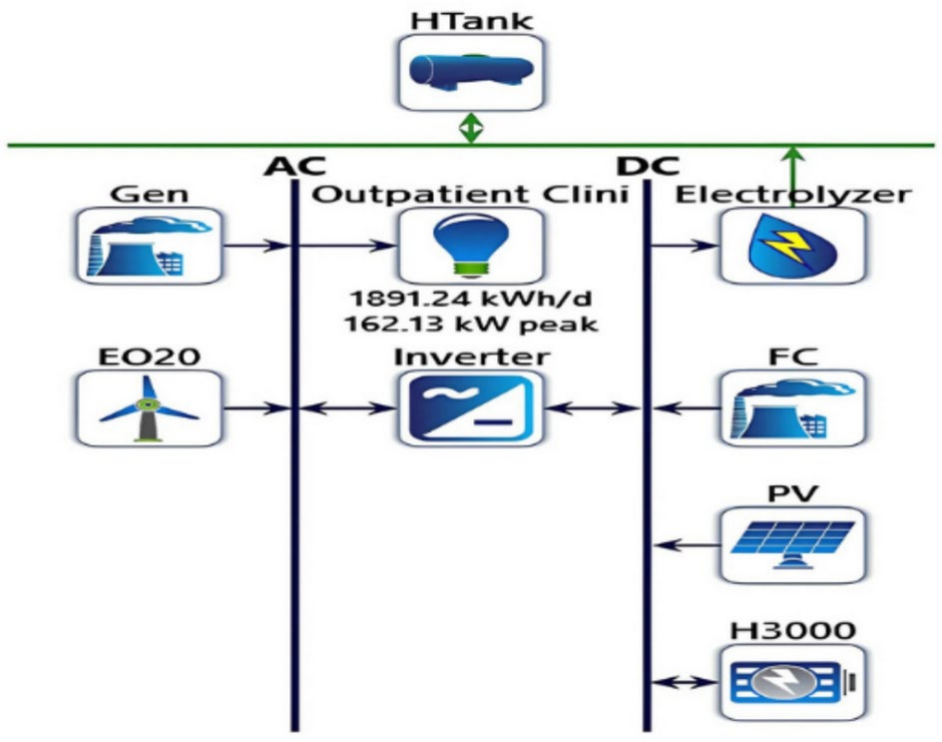
The optimized system architecture.

The plant site in 130 Street 11, New Cairo 1, Cairo Governorate, Egypt (29°59.8’N, 31°26.2’E), was optimized. This IMG has a max power of 162.13 kW and uses 1891.24 kWh daily. HOMER assesses the price and establishes the feasibility of HRES throughout the project. Simulations created ideal system configurations and their capabilities, which were selected according to the lowest TNPC and the LCOE. This study chose the optimum system architecture that optimally fits the configuration system. Figure 7 displays the system that is the most effective. Table 3 contains a summary of the optimum components system specification.

**Table 3:** Optimized components detail.

Component	Name	Size	unit
Generator #1	Fuel Cell	50.0	kW
Generator #2	Diesel Generator	180	kW
PV	PV	200	kW
Storage	Batteries (96 battery)	capacity = 96 × 7.15 = 686 kWh	4 strings
Wind Turbine	Eocycle EO20	9 × 20 = 180 kWh	kW
System Converter	Studer AJ 2400–24	141	kW
Electrolyzer	Electrolyzer	50.0	kW
Hydrogen Tank	Hydrogen Tank	50.0	kg
Unmet Electrical Load	0%	Excess Energy	33%
Dispatch Strategy	HOMER Load Following		

### Power production

**Fig. 8:**
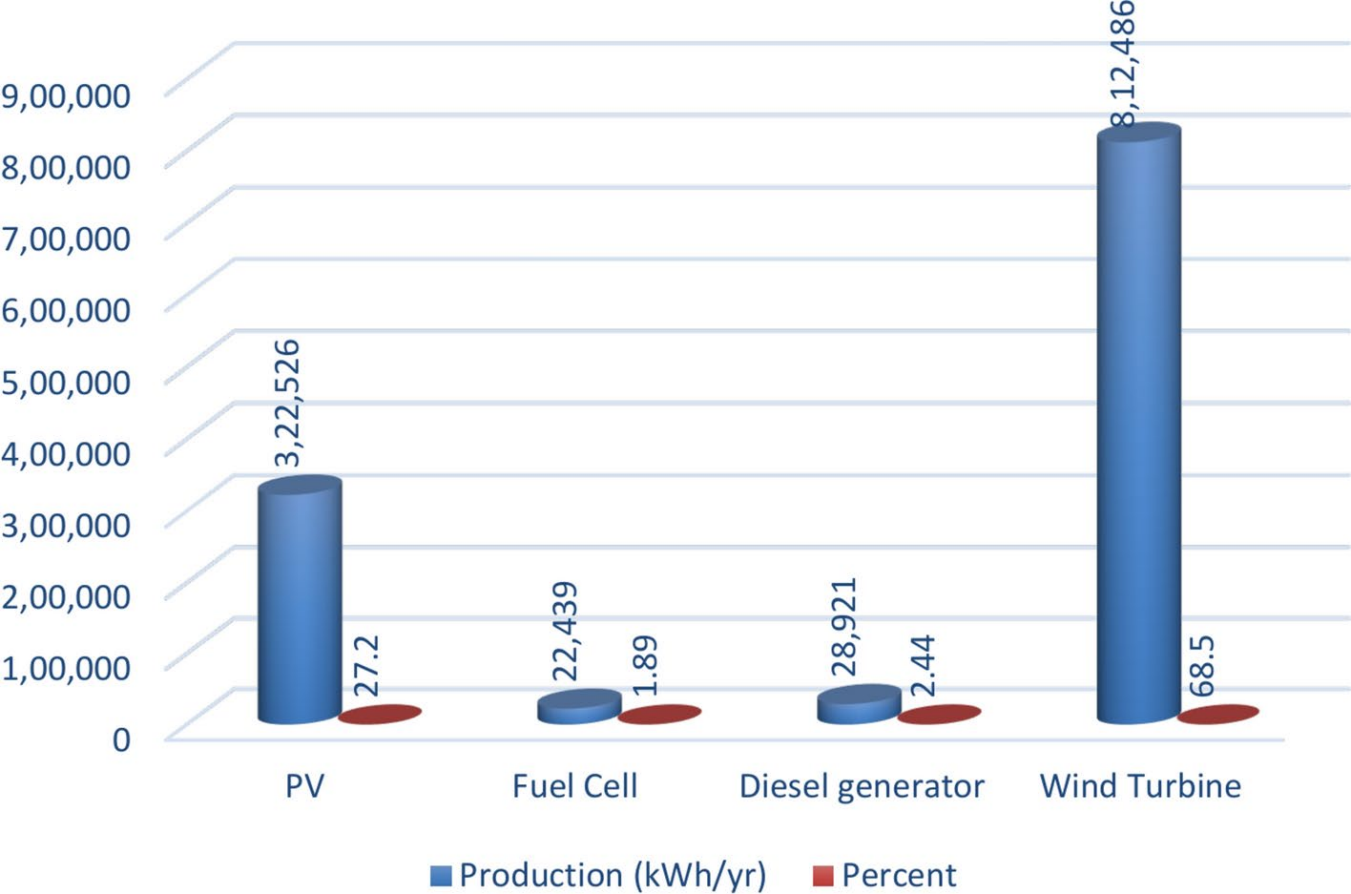
Overview of production for every component in the perfect power system.

**Fig. 9:**
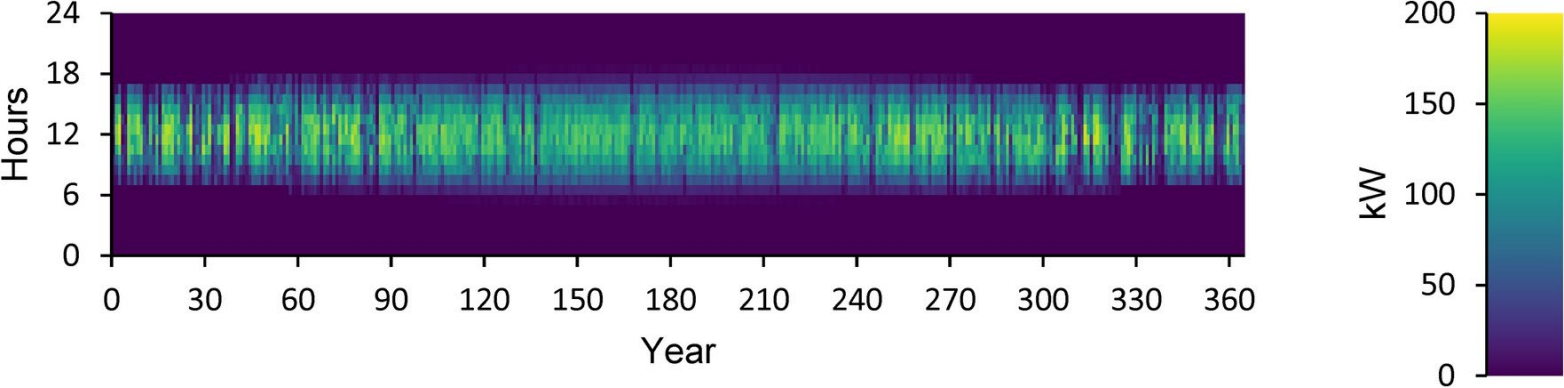
Yearly PV energy production during the full 24 h of an optimal system operation.

**Fig. 10:**
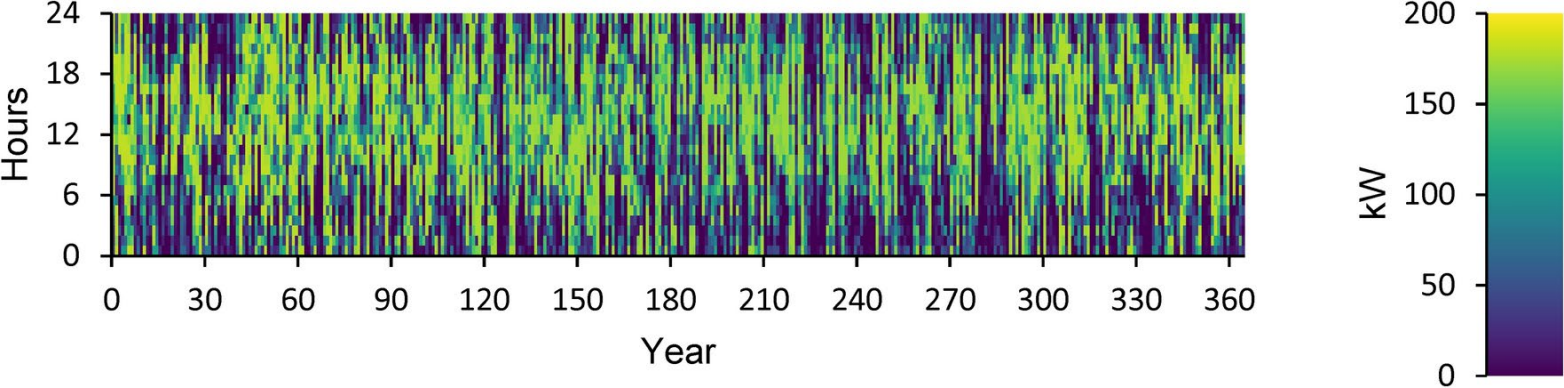
Yearly WT energy production during the full 24 h of an optimal system operation.

**Fig. 11:**
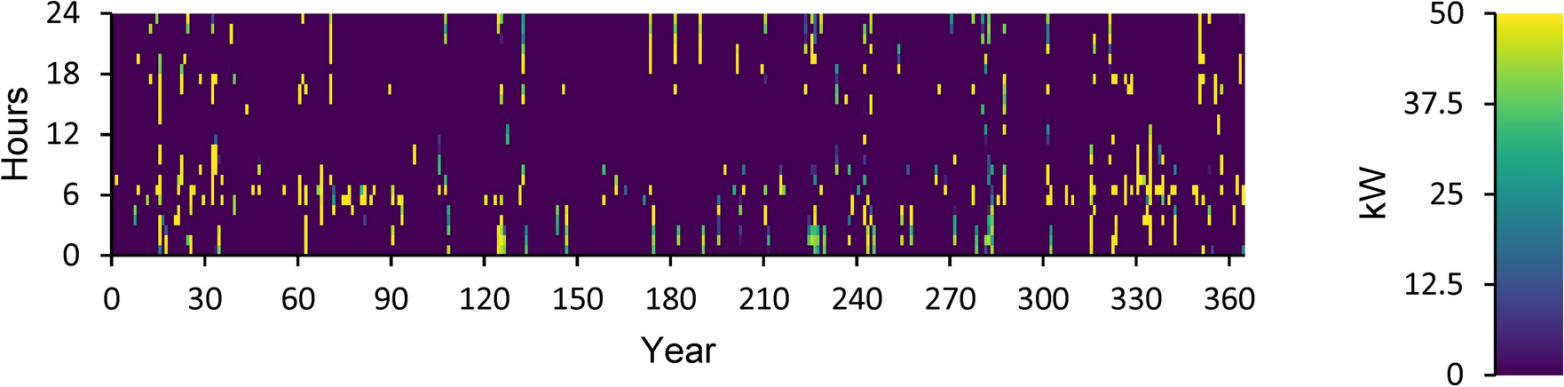
Yearly FC energy production during the full 24 h of an optimal system operation.

A power overview of the output and input information for every component of an optimal power system is displayed in Appendix A. The generation overview for every element of the optimized power system is shown in Fig. 8. In this investigation, the WT system produced the greatest quantity of power, accounting for 68.5% of the total power generated. A minimum of 1.89% of the total power is generated using FC. PV power generation accounts for 27.2% of the total power generated. DG generates 2.44% of the total power generated in electricity. The yearly power produced from PV during the optimized power system’s 24-h day is displayed in Fig. 9. This data indicates that the average power output is 36.8 kW and 186 kW at its maximum. The PV system generates an average of 884 kWh of energy each day and 322,526 kWh of energy annually. There are 4385 h of operation in total annually. The yearly energy generated from WT during a 24-h day in the optimized electrical system is displayed in Fig. 10. This figure indicates that the average power generation is 92.7 kW, while the maximum output energy is 182 kW. 812,486 kWh of electricity is produced annually by WT. There are 7,622 h of operation in total each year. The yearly electrical output from FC during the optimized power system’s 24-h day is displayed in Fig. 11.

**Fig. 12:**
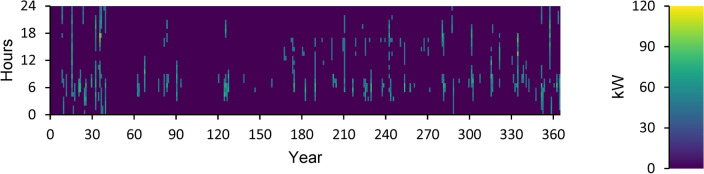
Yearly DG energy production during the full 24 h of an optimal system operation.

**Fig. 13:**
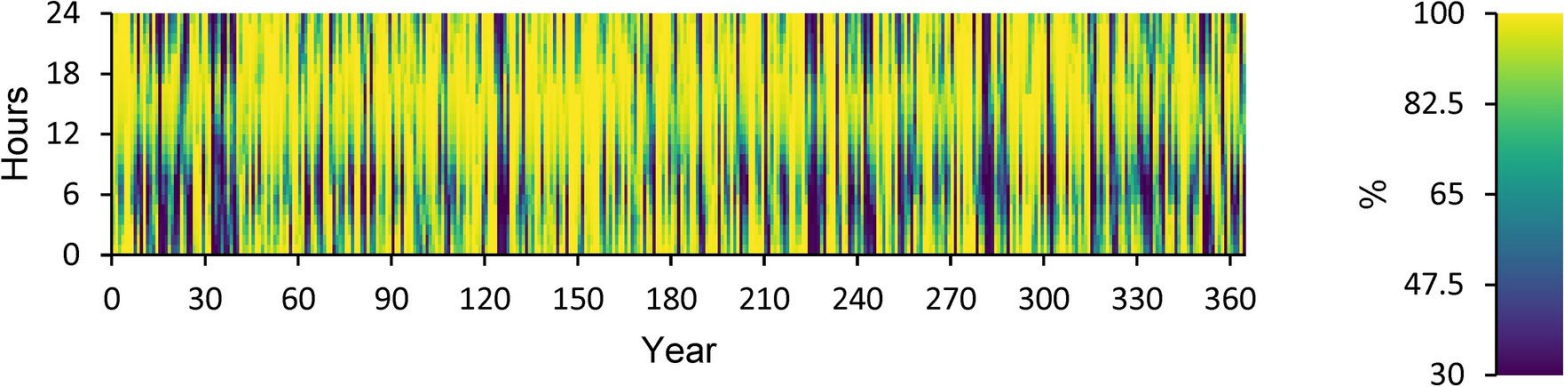
Yearly SOC (in percentage) of batteries during 24 h of an optimal system operation.

According to this, the fuel consumption is 1,346 kg, and the specific fuel consumption is 0.06 kg/kWh. The output power is observed to be 50 kW at maximum and 35.9 kW at average. The yearly energy generated from the DG during a day in the optimized power system is displayed in Fig. 12. This figure indicates that there are 548 h of operation annually. The output power is 52.8 kW on average and 108 kW at its highest. The DG has an operational life of 27.4 years and 146 starts yearly, respectively. The yearly SOC (in percent) of the LI Battery bank for the perfect structure throughout 24 h is shown in Fig. 13. This statistic indicates that the batteries are at 30% and 100% of their initial and least states of charge, respectively. Batteries have an annual energy input of 102,937 kWh and an annual energy output of 88,551 kWh. The annual energy loss is roughly 14,413 kWh. The throughput is 95,487 kWh annually.

**Fig. 14:**
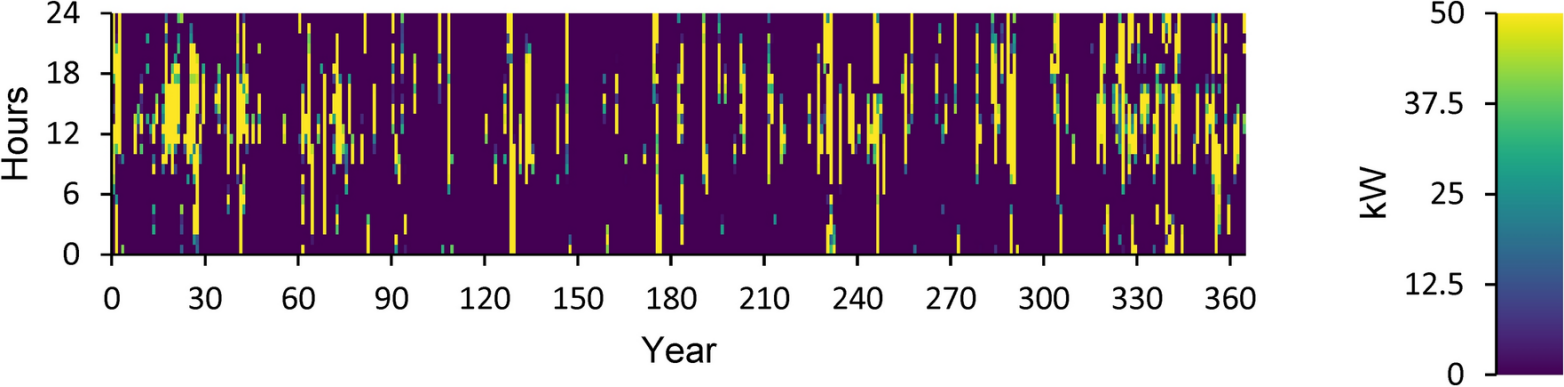
Yearly ELZ operation during the full 24 h of an optimal system operation.

**Fig. 15:**
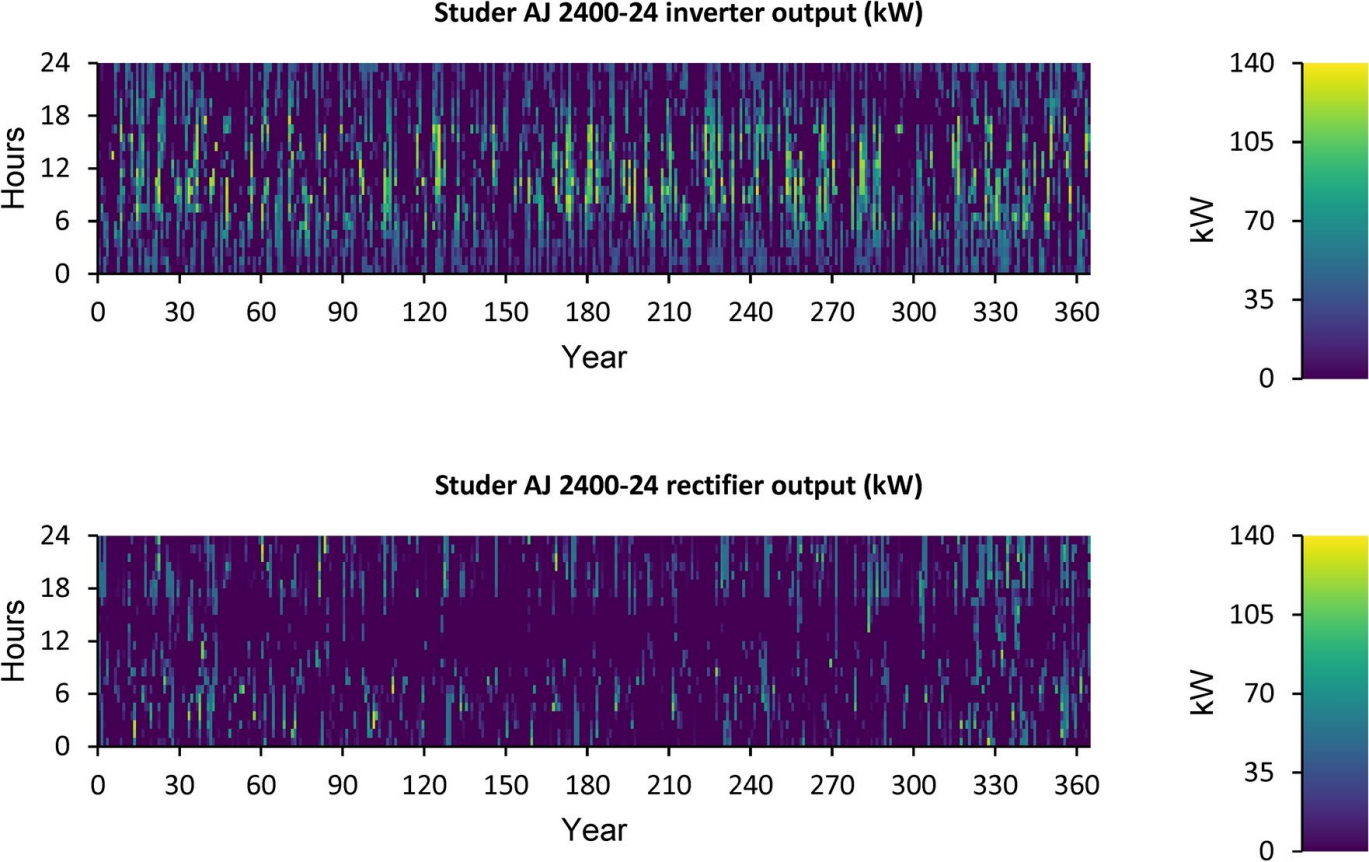
Yearly Converter operation during the full 24 h of an optimal system operation.

**Fig. 16:**
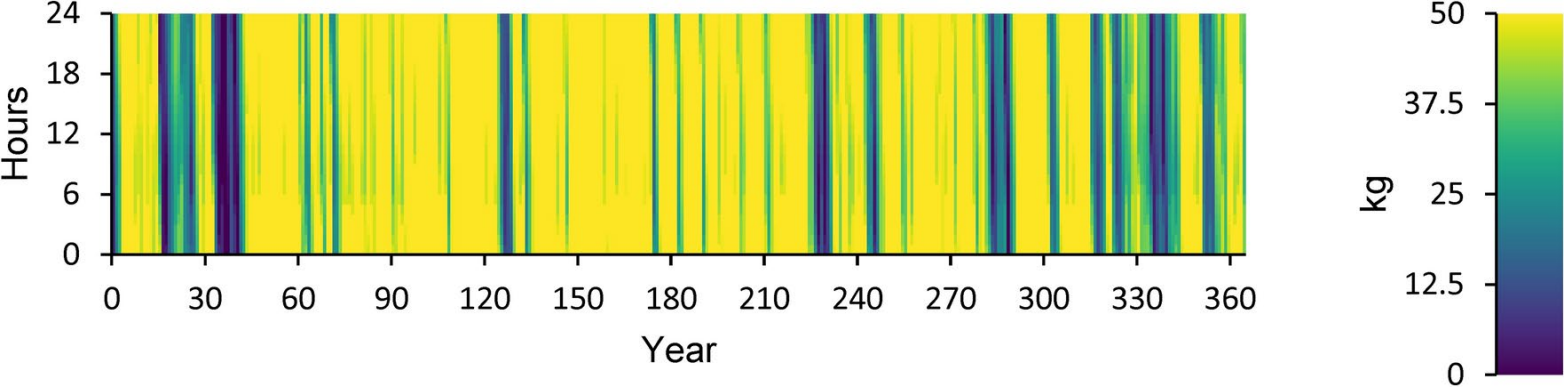
Yearly Hydrogen Tank SH Level operation during the full 24 h of an optimal system operation.

Figure 14 displays the ELZ’s annual operation, Fig. 15 illustrates the Converter’s annual operation, and Fig. 16 depicts the HT’s annual operation. All figures present the operational characteristics of these components over 24 h, showcasing their performance within a perfect system structure.

### Renewable power

**Fig. 17:**
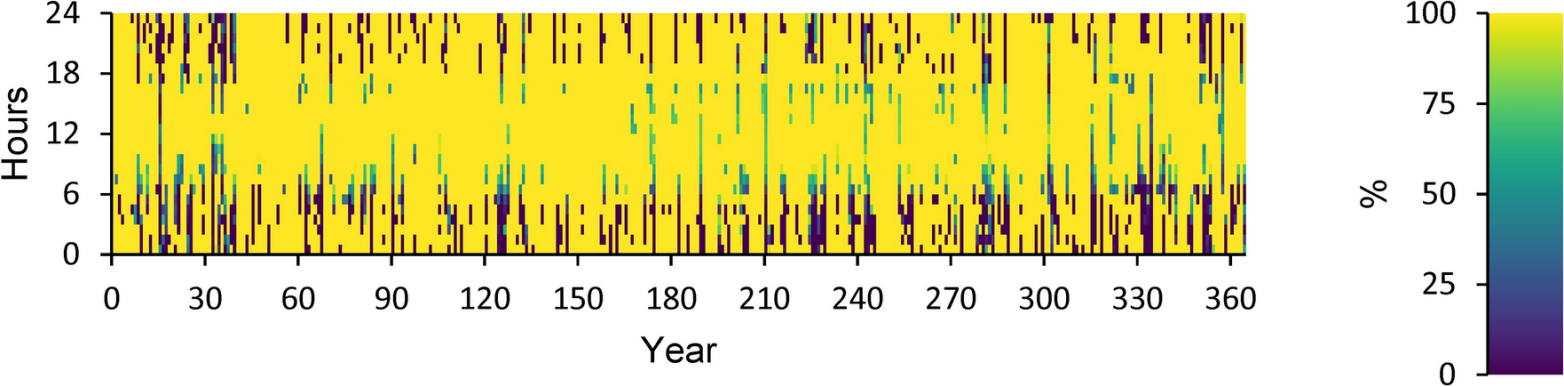
Renewable power as a percentage of total generation.

**Fig. 18:**
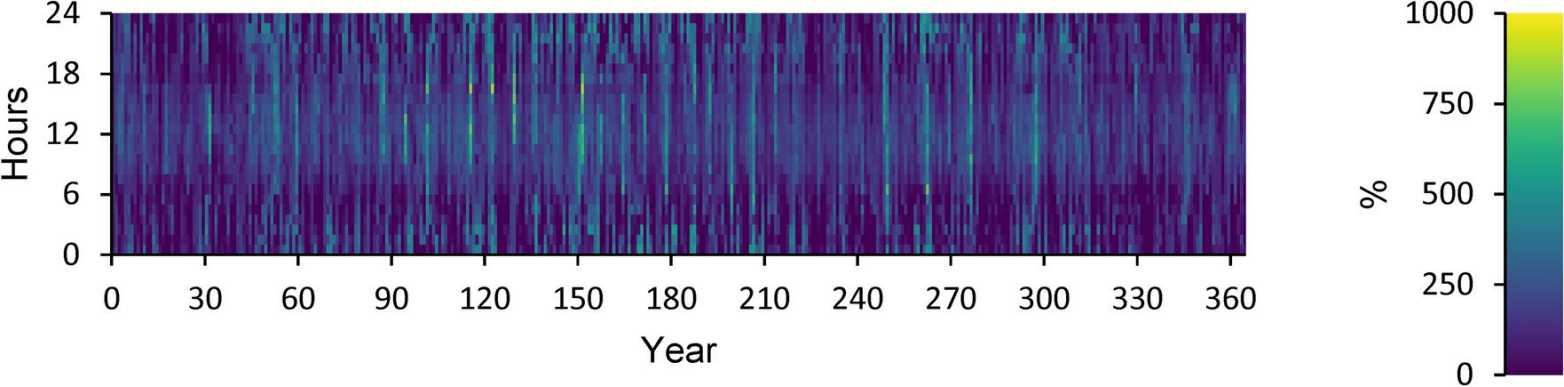
Renewable power as a percentage of total load.

**Fig. 19:**
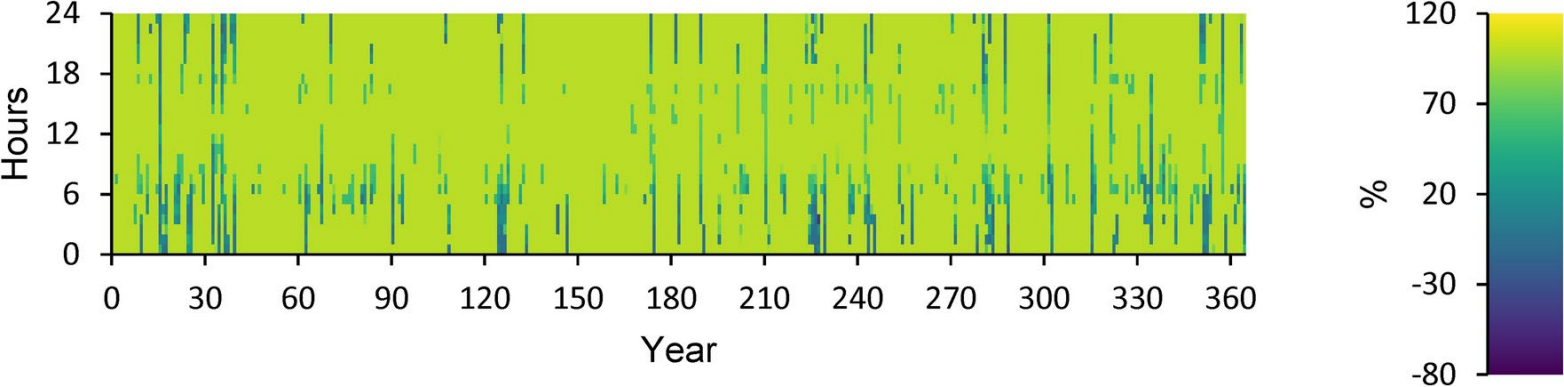
Non-renewable power is subtracted from 100% of the total annual load.

Table 4 shows several indicators concerning renewable energy, including capacity-based, energy-based, and peak values. Figure 17 displays Renewable power as a percentage of total generation. Figure 18 illustrates Renewable power as a percentage of the total load. Figure 19 depicts Non-renewable power subtracted from 100% of the total load over a year.

**Table 4:** Renewable summary.

Capacity-based metrics	the nominal capacity of renewables divided by the total nominal capacity	62.3%
	Utilizable renewable capacity divided by the total capacity	43.9%
Energy-based metrics	total renewable output divided by load	150%
	total renewable output divided by production	95.7%
	One minus total nonrenewable output divided by load	93.2%
Peak values	Renewable output divided by load (HOMER standard)	844%
	Renewable output divided by total production	100%
	One minus nonrenewable output divided by the total load	100%

### Economic analysis results

**Fig. 20:**
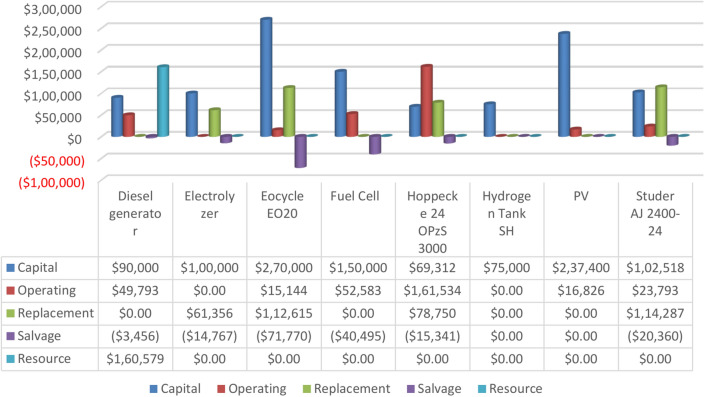
An overview of the optimal system’s costs.

The findings showed that the optimized system proposed in the study possessed the most favorable economic characteristics throughout the entire duration of the project. It was determined to have the ideal combination of system structure, incorporating PV, WT, FC, DG, and batteries. The TNPC of the system was determined to be USD 1,775,300.00, with an LCOE of 0.153 USD/kWh. Figure 20 provides a summary of the expenses related to the optimal system. This Figure presents detailed information on the capital cost, replacement cost, Resource costs, operating costs, and salvage costs for each component of the system. It also displays the overall price of every element and the system’s overall TNPC. Additionally, the Figure provides a detailed breakdown of the costs of every component in the perfect system. In summary, the cost breakdown shows that the WT component has the highest initial cost, the Hoppecke 24 OPzS 3000 battery has the highest operating cost, the Converter incurs the maximum replacement cost, the DG has the highest resource cost, the WT component has the highest salvage cost, and the WT component also has the highest total cost. Table 5 shows the costs of design optimization for our suggested islanded AC MG concept in Egypt compared to comparable local and international projects. Indirect comparability exists between the TNPC values for these models because of variations in component sizing and capital investments. Instead, we employ the LCOE as a critical tool to assess energy costs. The Table presents our results, demonstrating how well our model performs compared to previous research, with a competitive LCOE of $0.153/kWh.

**Table 5:** Comparative analysis between proposed optimal islanded AC MG model with literature studies.

Ref	Year	City, country	Optimal System Configuration	Economic Feasibility	
				TNPC ($)	LCOE ($/kWh)
^7^	2023	Najran, Saudi Arabia	PV/BESS	10,344	0.189
^8^	2022	Pradesh, India	PV/BESS	7.01 M	0.244
^9^	2021	Kayonza, Rwanda	PV/BESS	903,829	0.2
^10^	2020	Sahara Of Algeria	PV/BESS	23,427	0.23
^11^	2020	Xining, China	WT/BESS	58,178	1.247
^12^	2023	Iran	PV/WT/ BESS	50,417	0.692
^13^	2021	Odissa, India	PV/WT/ BESS	454,242	0.278
^15^	2020	Al-Batinah, Oman	PV/WT/ BESS	60,562	0.28
^16^	2020	Baluchistan, Pakistan	PV/WT/ BESS	28,620	0.311
^17^	2023	Shah Alam, Malaysia	PV/ BESS	3.25 M	0.184
^18^	2022	Chhattisgarh, India	PV/ DG/ BESS	1.71 M	0.379
^19^	2021	Kudu, Nigeria	PV/ DG/ BESS	16,457	0.259
^20^	2021	Andhra, India	PV/ DG/ BESS	341,280	0.217
^21^	2020	Pategi, Nigeria	PV/ DG/ BESS	1,079,015	0.403
^22^	2020	Fouay, Benin	PV/ DG/ BESS	555,492	0.207
^23^	2020	Sharjah, UAE	PV/ DG/ BESS	44,483,730	0.25
^24^	2020	United Kingdom	WT/DG/ BESS	14,507	0.588
^25^	2022	Bhola, Bangladesh	PV/WT/DG	141 M	0.691
^26^	2024	Myanmar- Bangladesh	PV/ DG /BESS	424,354	0.259
^27^	2024	Gwadar, Pakistan	PV/WT/DG /BESS	1.17 M	0.295
^28^	2023	Cameroon	PV/WT/DG /BESS	63,312	0.1691
^29^	2023	Perhentian, Malaysia	PV/WT/DG /BESS	387,185	0.64
^30^	2023	Abuja, Nigeria	PV/WT/DG /BESS	1,795,026	0.1616
^32^	2022	Andhra, India	PV/WT/DG /BESS	5.48 M	0.272
^33^	2022	Aceh, Indonesia	PV /DG /BESS	1.39 M	0.246
^34^	2020	West China	PV/WT/DG /BESS	587,013	0.201
^35^	2023	Neom, Saudi Arabia	PV/FC/HT/ELZ	703,194	0.498
^36^	2021	Gujarat, India	PV/WT/ FC/ HT/ELZ	1,638,635	0.3387
^37^	2022	Delhi, India	PV/WT/FC/BESS/HT/ELZ	1 519 040	0.264
^38^	2024	Iran	PV/WT/DG/BESS	180,065.43	0.32
^39^	2024	Oman	PV/FC/ HT/ELZ	317,401,96	0.97
^40^	2024	Technopark, Indonesia	PV/BESS	250,478	0.28
^41^	2024	Sub-Saharan Africa	PV/WT/FC/BESS/HT/ELZ	14,272	0.336
^42^	2024	Aïn El Ibel, Algeria	PV/WT/DG/BESS	376,063.8	0.2109
^43^	2024	Iran	PV/FC/ HT/ELZ	22,000	0.28
^44^	2024	Krishnanagar, India	PV/DG/BESS	35,627.85	0.248
^45^	2024	Kandahar, Afghanistan	PV/DG/BESS	10,108	0.190
^46^	2024	Ogun, Nigeria	PV/WT/DG/BESS	1782,602	0.26
^48^	2023	Alexandria, Egypt	PV/WT/BESS	125 M	0.158
^49^	2019	Sohag, Egypt	PV/ DG/BESS/ flywheel	28.5 M	0.200
^51^	2024	Aswan, Egypt	PV/WT/DG/BESS	853,634.53	0.255
^52^	2021	Hurghada, Egypt	PV/WT/DG/BESS	4.95 M	0.275
^53^	2019	Western Sahara, Egypt	PV/WT/DG/BESS	3,964,526	0.1682
^54^	2022	Red Sea, Egypt	PV/WT/DG/BESS	103 M	0.224
^55^	2020	Marsa-Matruh, Egypt	PV/WT/DG/BESS	351,223	0.2262
^56^	2020	Red Sea, Egypt	PV/WT/DG/BESS	3.12 M	0.164
^57^	2024	Cairo, Egypt	PV/WT/FC /HT/ELZ	8,119,087	0.2116
^58^	2023	Ras-Ghareb, Egypt	WT/FC/HT/ELZ	1.81 M	0.3085
Present study		New Cairo, Egypt	PV/WT/DG/FC/BESS/HT/ELZ	1,775,300	0.153

### Power system stability analysis

**Fig. 21:**
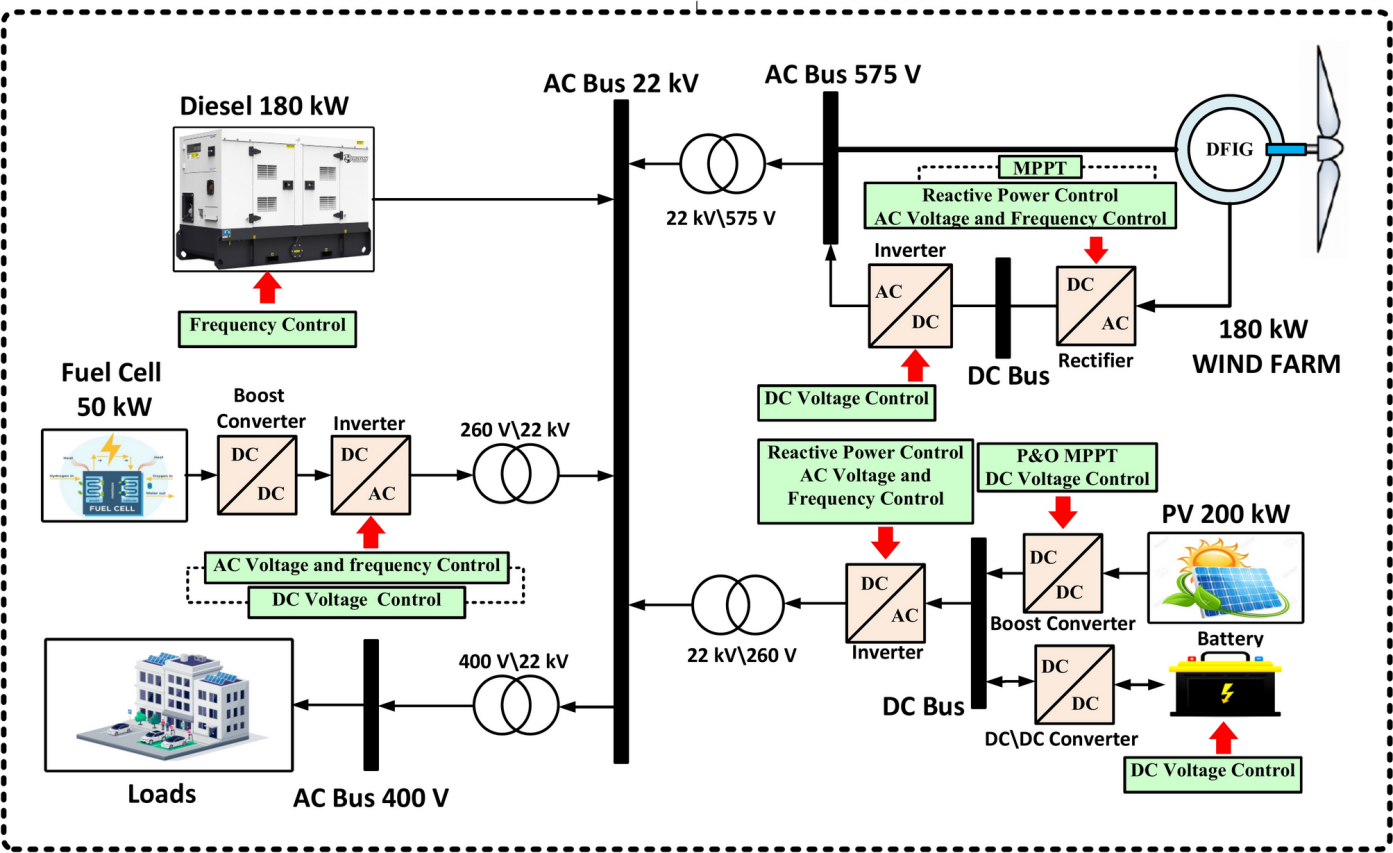
The proposed system structure.

Figure 21 illustrates the configuration of an islanded AC MG system. Components such as WT, PV, DG, FC, BESS, and Loads are connected to a 22 kV AC bus (primary distribution voltage) through electric converters. The wind farm’s double-fed induction generator (DFIG) has nine 20-kW turbines. DFIG’s wound rotor connects to the grid through two AC-DC-AC converters. Generated electricity is stored in a DC-link capacitor. Converters maintain constant output frequency despite wind speed changes. Control systems manage input power, reactive power, and grid voltage. DFIGs stator and AC bus link via a step-up transformer. PV system delivers power at proper voltage using a boost converter, the PV array converts solar radiation to DC, the Inverter converts DC to AC for MG transfer, and the AC voltage is increased via a transformer to match grid voltage. FC unit boosts MG power generation by converting chemical energy to electrical power using oxygen and hydrogen. Low DC voltage is increased using a boost converter. RES is the primary power resource of the MG. Knowing that RES power is relative to the site’s resource data. Therefore, a lesser fluctuation in RES power may result in instability of the MG. The DG serves as a backup in the system and consists of the governor, excitation system, and synchronous machine, forming the DG for the diesel engine. Batteries are essential for maintaining network stability and storing additional energy, and the MG powers AC loads using an inverter linked to the DC bus. Parameters of the islanded AC MG are given in Table 6.

**Table 6:** Parameters of MATLAB simulation components.

Section	Parameters	Symbol	Value	Section	Parameters	Symbol	Value
WT	Rated power	PWT	20 kW	PV	Rated power	PPV	200 kW
	Rated voltage	VWT	575 V		No. of series cells	Ns	7
	Rated frequency	Fr	50 Hz		No. of parallel cells	Np	69
	Rated speed	${\text{v}}_{\text{rated}}$	13 m/s		Microgrid voltage	VBUS	22 kV
FC	Rated power	PFC	50 kW	DG	Rated power	PDG	180 kW
	Rated voltage	VFC	330 V		Rated voltage	VDG	400 V
	Type	PEM			Pole pairs	p	1
BESS	Rated capacity	PC	2286 Ah	Load	450 kW		
	Rated voltage	VBESS	300 V				
	Initial state-of-charge (%)	SOC	60%		Power factor		0.9

### Load frequency control

The objective of LFC is to maintain a power system’s power balance by adjusting the system frequency in response to changes in actual power demand. DG is typically used to build IMGs as a steady power source for correcting the fluctuating power from RES and load demand, as the frequency balance is quickly disrupted when MGs are separated. Additionally, the BESS is included in this IMG as an auxiliary regulation. This study focuses on an islanded AC MG with various sources such as WT, PV, DG, and FC. Power electronic components convert DC voltages from sources like PV arrays and FC into AC and connect them to the AC bus. Additionally, a converter is considered for the BESS system to convert AC to DC during charging and DC to AC during discharging. The power output of DG, WT, PV, FC, and BESS collectively represents the total power generated to meet the demand.

**Fig. 22:**
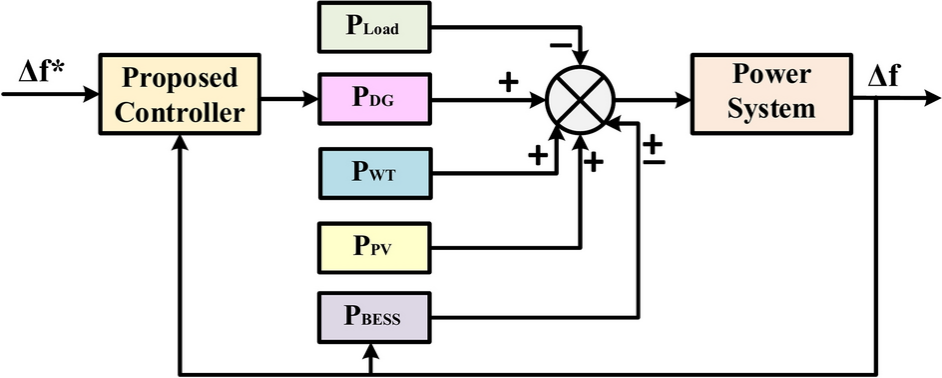
The block diagram of the microgrid control system is based on the proposed controller.

This study focuses on utilizing DG for frequency control, as electricity generated by PV, WT, and FC is not typically used for this purpose. In a frequency control loop, changes in the output powers of loads, WT, PV, and FC are compensated by adjusting the DG’s output powers, as displayed in Fig. 22. The frequency error is amplified, combined, and transformed to generate a command signal for the turbine governor. The governor then adjusts the turbine output to restore the balance between input and output. The frequency deviation (Δf) is determined by the difference in active powers between consumption and production (ΔP). Power management and frequency control are accomplished when the controller achieves zero frequency deviation (Δf).

## Control strategy

The paper focuses on evaluating the effectiveness of PI-PSO, PI-WOA, and MRAC-PI controllers in controlling the system’s frequency. The article aims to provide insights and a comparative analysis of these control strategies for improved operation and stability in MG.

### PI-PSO Controller

Evaluation of the PI controller’s efficiency in keeping up with the system’s frequency and improving the stability and functionality of the MG is given special attention. Equation (35) is used by the PI controller to determine the control signal (D) for the converter. The error signal’s measured frequency (${F}_{m}$), reference frequency (${F}_{ref}$), and sampling time (${T}_{s}$) are all included in the calculation^64^.

35 $$D=\left({F}_{ref}-{F}_{m}\right)\left({K}_{p}+ {K}_{i}\frac{{T}_{s} Z}{Z-1}\right)$$

**Fig. 23:**
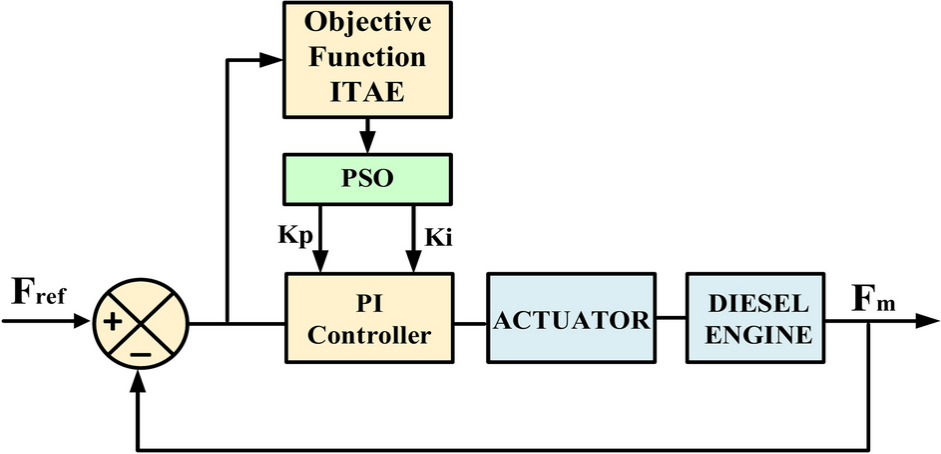
Structure of a PI-PSO controller-based closed-loop control system.

Additionally, Fig. 23 displays the block diagram of the closed-loop control system, which includes the PI-PSO controller. The PI controller’s gains (Kp and Ki) are adjusted using PSO. By formulating frequency control as an optimization task, it becomes possible to reduce the "integral of time multiplied by absolute error (ITAE)" performance measure^65^.

36 $$ITAE= {\int }_{0}^{T}t \left|e\left(t\right)\right|d(t)$$

where (t) is the time and e(t) is the difference between ${F}_{m}$ and ${F}_{ref}$.

Researchers favor the PSO technique’s simplicity and reliance solely on a fitness function. Numerous studies validate its effectiveness in various optimization scenarios, such as integer programming, minimum–maximum problems, and multi-objective optimization. However, the standard PSO can decrease population diversity during communication, which may restrict solutions to local optima rather than achieving global optimization^66^. Here are three unique characteristics of PSO^67^:

- ${P}_{pest}$ : The highest fitness value achieved by particle (i) up to this point.
- ${g}_{pest}$: The best fitness value is reached by any particle in the swarm.
- Velocity and Position Updates: Employed to navigate the search space in pursuit of the optimal solution.

**Fig. 24:**
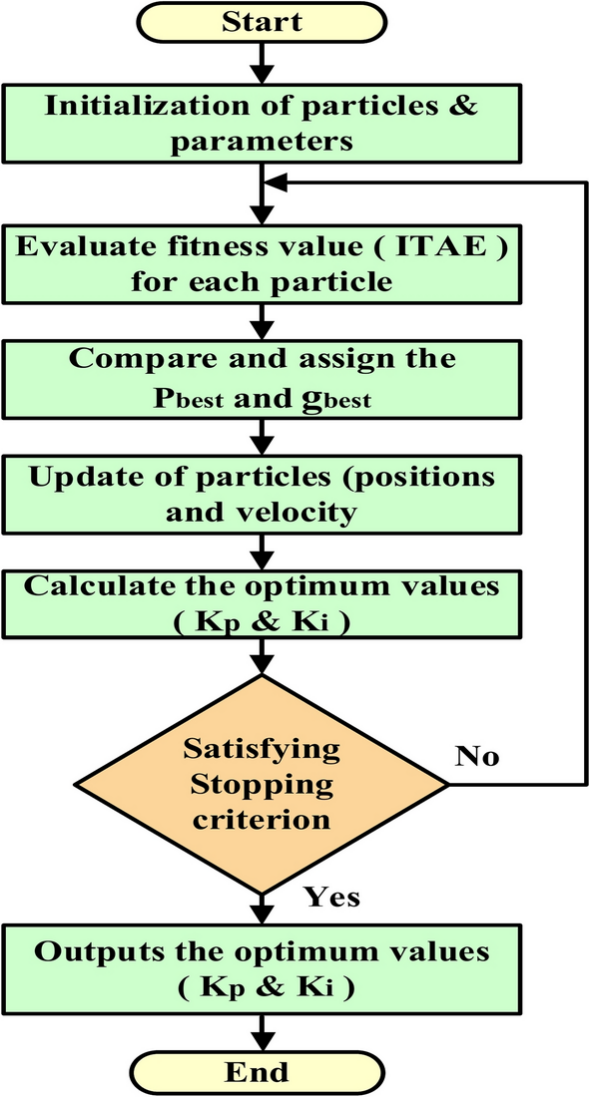
PSO flowchart.

The velocity of a particle is updated according to Eq. (37), where Eq. (38) presents how the particle’s new position is calculated by adding its new velocity to its previous position^68^:

37 $${v}_{id}\left(1+t\right)={ wv}_{id}\left(t\right)+{ r}_{1}{C}_{1}\left({ P}_{pest,id}\left(t\right)- { X}_{iid}\left(t\right) \right)+ { r}_{2}{C}_{2}\left({ g}_{pest,id}\left(t\right)- { X}_{id}\left(t\right) \right) d=\text{1,2},\dots .D$$

38 $${X}_{id}\left(1+t\right)={{ v}_{id}\left(1+t\right)+ X}_{id}\left(t\right) d=\text{1,2},\dots .D$$

where, ${(P}_{pest,id})$ represents the best previous position of particle (i), while ${(g}_{pest,id})$ indicates the best global position for the particle. The variable $(w)$ refers to the inertia weight, $(C)$ is the learning rate, and (*r*) is a randomly chosen value. This equation consists of three elements: social, cognitive, and inertia. The term ${(wv}_{id})$ reflects the inertia component, which maintains the influence of past movements and directs the particle’s progress at iteration (t). ${(C}_{1})$ represents the cognitive term, guiding particles to their best previous positions, whereas ${(C}_{2})$ signifies the social component, which evaluates the performance of particles and the swarm’s trajectory in the optimization space. Figure 24 presents the flowchart of the PSO algorithm^69^.

### PI-WOA Controller

**Fig. 25:**
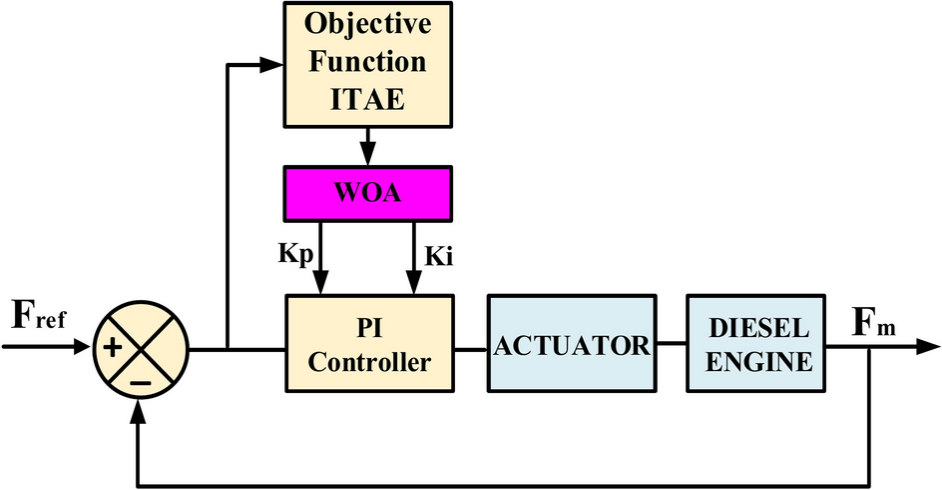
Structure of a PI-WOA controller-based closed-loop control system.

The Whale Optimization Algorithm (WOA) is an effective metaheuristic inspired by humpback whale behavior, excelling in complex optimization challenges. Its strength lies in balancing exploration and exploitation, resulting in faster convergence and better outcomes than traditional algorithms. WOA has proven successful in various applications, particularly in control system optimization^70^. In^71^, WOA-based MPPT control for proton exchange membrane fuel cells (PEMFC) efficiently tracks optimal output power during water content fluctuations, outperforming PSO, fuzzy logic control (FLC), and Perturb and Observe (P&O) methods. Additionally, the fuzzy-fractional Order PID (FFOPID) controller, enhanced by a WOA-centered Active Half Car Driver model, significantly reduces driver vibrations compared to fractional Order PID (FOPID) and PSO-tuned FFOPID controllers^72^. The WOA-optimized Fractional-Order Proportional Integral and Integral-Order Controller (FOPIλ) excels in solar PV-fed sensorless speed control of PMBLDC motors, surpassing the Bat Algorithm (BA) and Grey Wolf Optimization (GWO) by minimizing performance errors and convergence time^73^. Moreover, WOA-based PID controllers deliver quicker responses, less steady-state error, and improved damping compared to GA and Artificial Bee Colony (ABC) methods in modern power systems^74^. Figure 25 illustrates the speed control loop of the proposed PI-WOA controller-based closed-loop control system applied to control the rotor speed of the generator.

**Fig. 26:**
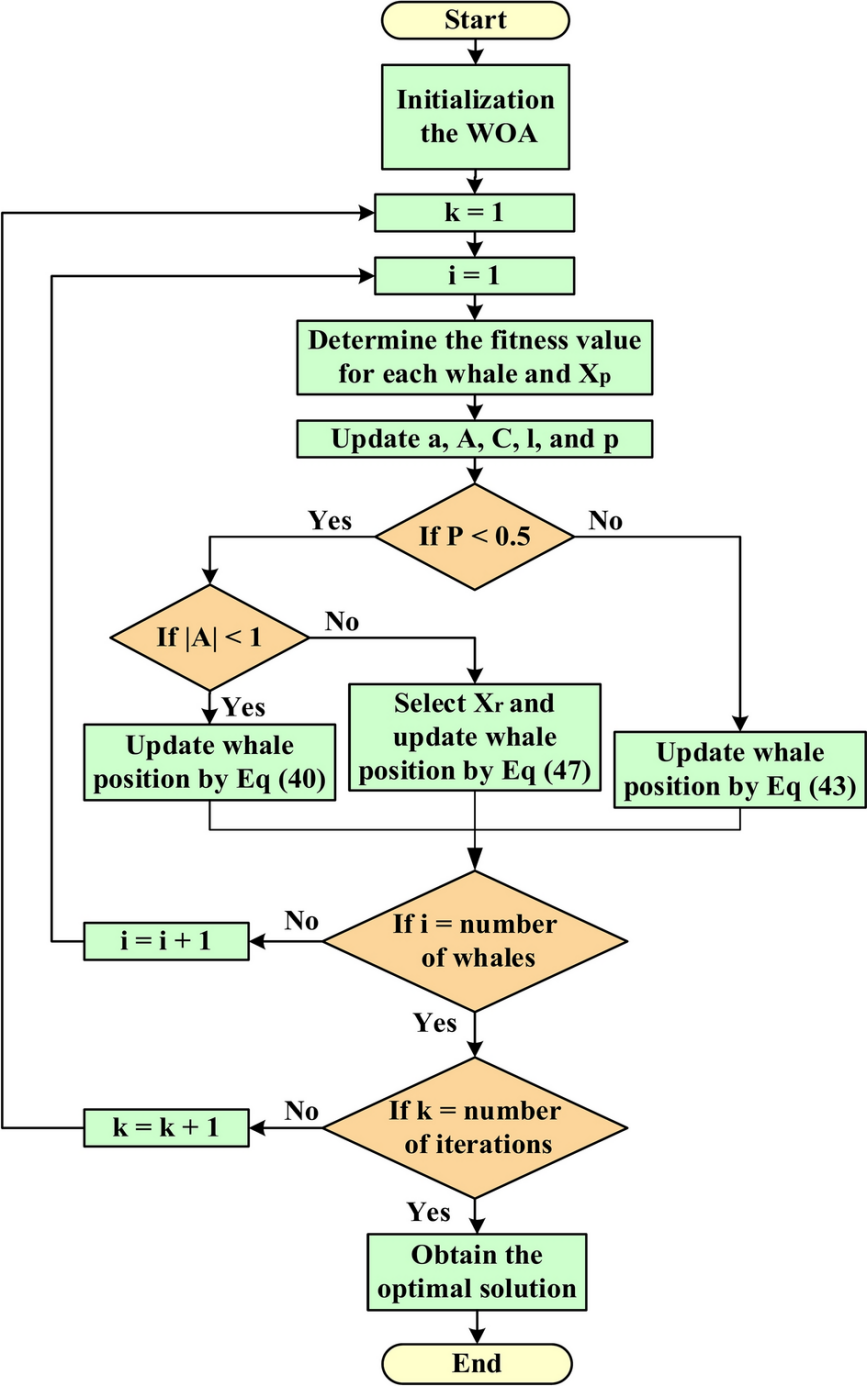
WOA flow chart.

Figure 26 displays the WOA flow chart. It can be represented by the equations below^75,76^:

39 $$\overrightarrow{D}=\left|\overrightarrow{C} . \overrightarrow{{X}_{P}}\left(t\right)-\overrightarrow{X}\left(t\right)\right|$$

40 $$\overrightarrow{X}\left(t+1\right)= \overrightarrow{{X}_{P}}\left(t\right)-\overrightarrow{D} .\overrightarrow{A}$$

41 $$\overrightarrow{A}=2\overrightarrow{a} .\overrightarrow{r} -\overrightarrow{a}$$

42 $$\overrightarrow{C}=2 . \overrightarrow{r}$$

43 $$\overrightarrow{X}\left(t+1\right)= \overrightarrow{{X}_{P}}\left(t\right)+\overrightarrow{{D}{\prime}}. {e}^{bl}.\text{cos}\left(2\pi l\right)$$

44 $$\overrightarrow{{D}{\prime}}= \left|\overrightarrow{{X}_{P}}\left(t\right)- \overrightarrow{X}\left(t\right)\right|$$

45 $$\overrightarrow{X}\left(t+1\right) \left\{\begin{array}{c}\overrightarrow{{D}{\prime}} . {e}^{bl}.\text{cos}\left(2\pi l\right)+\overrightarrow{{X}_{P}}\left(t\right) if p \ge o.5 \\ \overrightarrow{{X}_{P}}\left(t\right)-\overrightarrow{A} .\overrightarrow{D} if p <o.5\end{array}\right.$$

46 $$D=\left|{X}_{r}\left(t\right). \overrightarrow{C} X\left(t\right)\right|$$

47 $$\overrightarrow{X}\left(t+1\right)= {X}_{r}\left(t\right)-\overrightarrow{A} .\overrightarrow{D}$$

The symbols X(t), ${X}_{p}\left(t\right)$, and ${X}_{r}\left(t\right)$, corresponding to the position vectors of the whale, prey, and random whale, respectively. The letter (t) stands for the current iteration. The letters A and C stand for the coefficient vectors. Over the number of rounds, (a) constantly decreases linearly from 2 to 0. The random integer (l) is between − 1 and 1, the random vector (r) is between 0 and 1, the p is the probability number ε [0, 1], and the constant that determines the spiral logarithmic form is represented by (b).

**Fig. 27:**
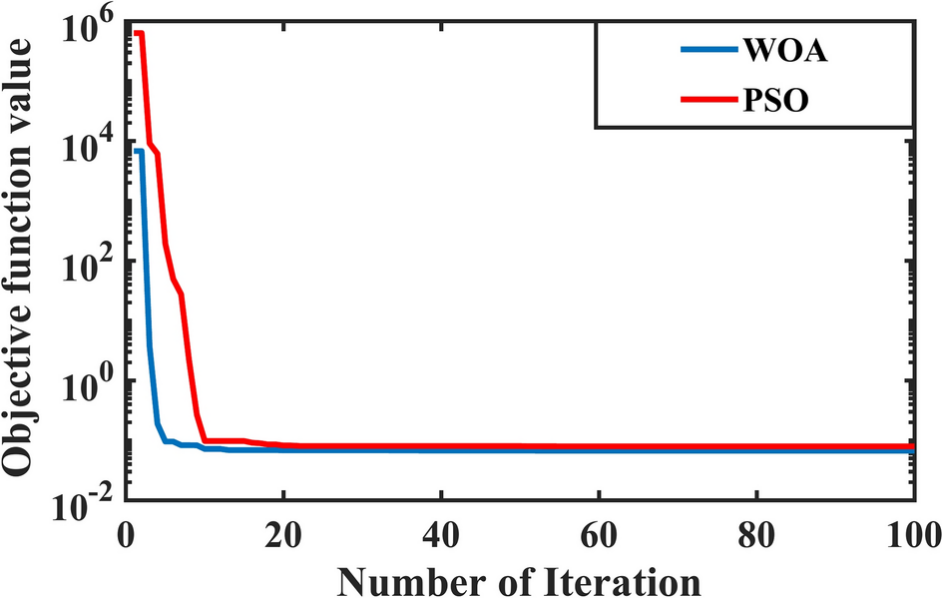
Convergence of the objective function.

Figure 27 points out the convergence of the objective function for the two optimization algorithms. The parameters of optimization algorithms and the optimal gains of the PI controllers using the PSO and WOA are shown in Table 7.

**Table 7:** The parameters of WOA and PSO.

Optimization Algorithms			
WOA		PSO	
Parameter	Value	Parameter	Value
No. of whales (Population size)	50	No. of particles (Population size)	50
Max Iteration	100	Max Iteration	100
reduced value for the coefficient values(a)	0 < a < 2	Cognitive component (C1)	1.5
The random integer (l)	-1 < I < 1	Social component (C2)	1.5
Uniformly distributed random number(r)	0 < r < 1	Maximum inertia weight	0.4
P-value (used to determine the stage)	0.5	Minimum inertia weight	0.9
Lower bound	[ 0, 0 ]	Lower bound	[ 0, 0 ]
Upper bound	[ 1500, 1500 ]	Upper bound	[ 1500, 1500 ]

### MRAC-PI Controller

**Fig. 28:**
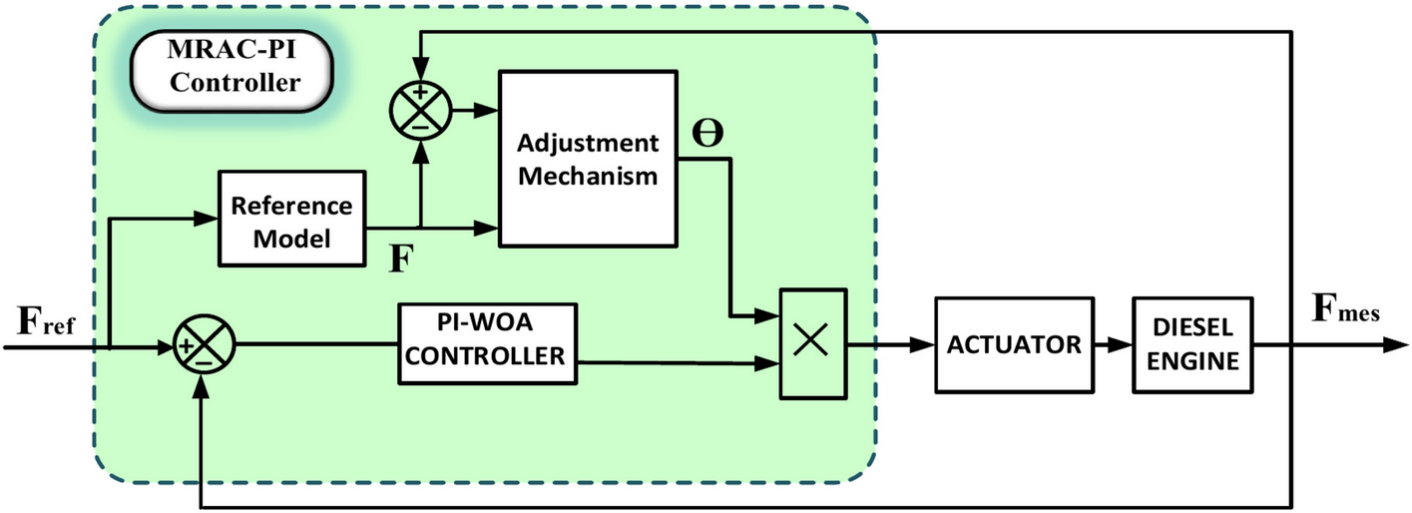
Structure of a MRAC-PI controller-based closed-loop control system.

To enhance the accuracy of the PI-WOA controller, we integrated it with Model Reference Adaptive Control (MRAC) to improve efficiency and facilitate adaptation. The use of MRAC in IMG provides substantial advantages, particularly in handling the variability of RES. MRAC enhances frequency stability and voltage regulation by making real-time adjustments to generation and load fluctuations, improving dynamic performance through continuous modification of control parameters based on system behavior. This leads to a more reliable and resilient energy supply, facilitates the integration of various RES, and increases the overall efficiency of IMG. Notable applications include a fractional order MRAC control law for stabilizing current and voltage in multi-source systems with DC-DC converters^77^, a modified MIT rule-based MRAC for DC-DC boost converters^78^, a strategy for managing the unified interphase power controller (UIPC) in hybrid MGs^79^, and the enhancement of traditional droop control in ship power systems^80^, as well as regulating the output voltage of DC-DC converters in isolated MGs^81^. As shown in Fig. 28, the MRAC-PI controller comprises three primary parts: the PI-WOA controller explained later, the adjustment mechanism, and the reference model.

A transfer function with discrete time ${G}_{r}$ is represented the closed-loop system’s intended behavior, which in this study is a zero overshoot and a 0.05 s settling period. and is modulable as:

48 $${G}_{r }\left(z\right)=\frac{(0.01z-0.0099)}{{z}^{2}-1.9801z+0.9802}$$

The Massachusetts Institute of Technology (MIT) regulation states that the cost function is described as^82^:

49 $$J\left(\theta \right)\frac{1}{2}= {({F}_{m}-F)}^{2}= \frac{1}{2} {{e}_{r}}^{2}$$

where ${F}_{ref}$ is the reference model’s output, θ is the adaptation parameter, and ${F}_{m}$ is the system’s overall output. Based on the type of disruption, the model reference output frequency is selected as a decaying exponential increase from the lowest frequency that is meant to be tracked to the intended value of 50 Hz. The MIT rule calls for minimizing the cost function when modifying the parameter θ. To achieve this, either move J’s negative gradient in the direction of the parameter θ, or

50 $$\frac{d\theta }{{dt}} = \gamma^{\prime } \frac{\partial J}{\partial } = - \gamma^{\prime } e_{r} \frac{{\partial e_{r} }}{\partial \theta } = - \gamma^{\prime } We_{r}$$

Considering the variations between the reference model and the system’s output, the adaptation mechanism modifies the control action as

51 $$\theta =-\left( {F}_{m}- F \right)F\gamma \frac{{T}_{s }z}{z-1}$$

The equation for control is :

52 $$D=\theta {u}_{f}$$

### MATLAB simulation results and discussions

The system, Fig. 21, will be tested through several situations to assess the efficacy and performance of the suggested control and to confirm that the proposed controller can stabilize AC bus voltage, achieving active power balance among the MG components, ensuring reliable AC voltage quality, and restoring the frequency of MG to the appropriate value after various disturbances, including load changes, changes in solar irradiance, and fluctuations in wind speed. The eight different disturbances are significant because they allow a thorough evaluation of the effectiveness and performance of the suggested control system for active power balancing among MG components and AC bus voltage stabilization. Every scenario illustrates typical challenges when integrating various energy sources into an MG. Through the analysis of these various disturbances, the suggested control solutions show how effective they are at preserving consistent AC voltage quality and ensuring the MG can quickly adjust to operational issues in the real world, improving overall system stability and resilience.

## CASE 1—A step change in solar irradiance

**Fig. 29:**
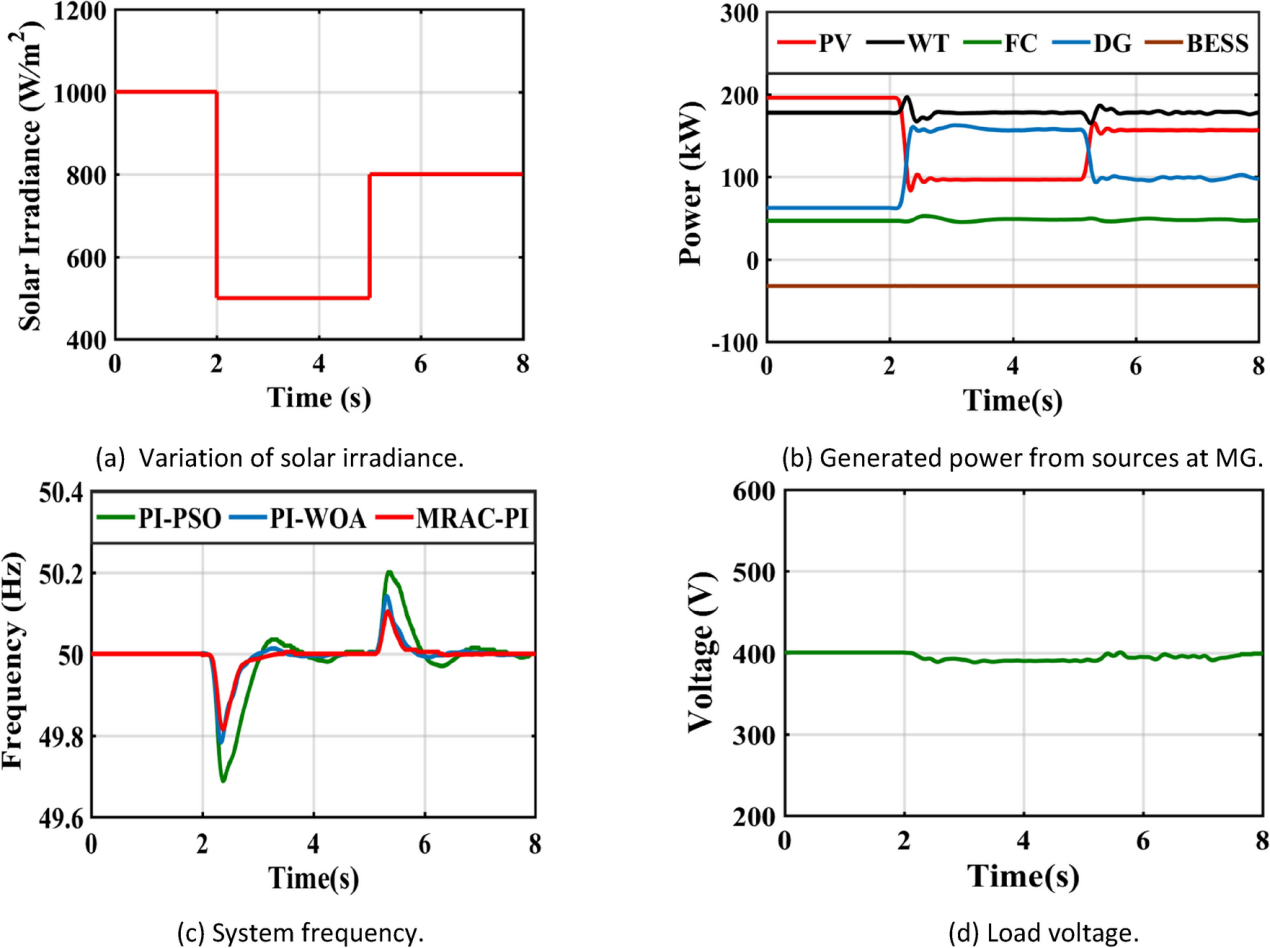
System response under case 1.

Figure 29(a) depicts the step variations in solar radiation intensity. It is important to understand that changes in solar radiation can significantly influence the microgrid’s frequency. The MRAC-PI controller is essential for maintaining effective frequency regulation under these conditions. In comparison to PI-PSO and P-WOA controllers, the MRAC-PI controller exhibits superior accuracy in responding to sudden changes in solar radiation, particularly during high-efficiency periods and rapid weather shifts. System performance is assessed using the MRAC-PI, PI-PSO, and PI-WOA controllers, focusing on critical metrics such as settling time (Ts), Integral of Time-weighted Absolute Error (ITAE), maximum overshoot (%Mp), and maximum undershoot (%Mus). Table 8 and Fig. 29(b) illustrate the MG frequency response following a step change. Figure 29(c) shows the output power from sources at MG, when the solar irradiance decreases from 1000 to 550 W/m^2^ at 2 s, the power produced by the PV declines from 196 kW to approximately 97 kW. To compensate for this power deficiency, the generated power from the DG resulted in a decrease in the rotor speed. It is possible to return the rotor speed to its synchronous value once it has decreased by increasing the mechanical power input to the DG. Subsequently, following frequency adjustment, the DG power rises from 63 kW to approximately 157 kW. In this instance, the FC continues to provide its maximum output while the WT runs at its ideal speed to generate the most power feasible. Conversely, when the solar irradiance rises from 550 to 800 W/m^2^ at 5 s, the power generated from the PV increases from 97 kW to approximately 157 kW, leading to a decrease in the generated power from the DG from 157 kW to approximately 98 kW. The MRAC-PI controller effectively maintains MG frequency stability despite these solar radiation fluctuations. Finally, Fig. 29(d) provides additional information showing the system’s voltage levels.

**Table 8:** The system response summary for each of the several suggested controllers in each scenario.

Cases		Controller type	System Frequency			
			Ts	ITAE	%Mp	%Mus
Case 1: A step change in solar irradiance	When solar radiation changes from 1000 (W/m^2^ ) to 550 (W/m^2^ )	PI-PSO	2.903	0.184	0.072	0.623
		PI-WOA	2.607	0.182	0.026	0.446
		MRAC-PI	1.688	0.103	0.010	0.371
	When solar radiation changes from 550 (W/m^2^ ) to 800 (W/m^2^ )	PI-PSO	2.925	0.203	0.401	0.062
		PI-WOA	2.827	0.194	0.284	0.016
		MRAC-PI	2.096	0.110	0.211	0.007
Case 2: A step change in wind speed	When wind speed changes from 13 to 11 m	PI-PSO	2.415	0.176	0.030	0.404
		PI-WOA	2.385	0.172	0.022	0.261
		MRAC-PI	1.585	0.101	0	0.195
	When wind speed changes from 11 to 12 m	PI-PSO	2.378	0.191	0.191	0.025
		PI-WOA	2.037	0.186	0.140	0.016
		MRAC-PI	1.267	0.104	0.070	0.001
Case 3: A sudden change in load	Total load demand decreased by 11.1% from 450 to 400 kW	PI-PSO	2.948	0.181	0.296	0.012
		PI-WOA	2.371	0.172	0.232	0.006
		MRAC-PI	2.103	0.109	0.170	0.005
	Total load demand increased by 6.25% from 400 to 425 kW	PI-PSO	2.615	0.186	0.011	0.130
		PI-WOA	2.215	0.180	0.006	0.110
		MRAC-PI	1.651	0.112	0	0.074
Case 4: Ramp changes in solar radiation		PI-PSO	5.496	0.218	0.190	0.285
		PI-WOA	5.230	0.217	0.106	0.160
		MRAC-PI	4.681	0.121	0.060	0.108
Case 5: Ramp changes in wind speed		PI-PSO	5.437	0.214	0.120	0.181
		PI-WOA	4.874	0.207	0.046	0.104
		MRAC-PI	4.430	0.117	0.031	0.066
Case 6: Random changes in solar radiation		PI-PSO	ــــــــــــ	0.245	0.341	0.330
		PI-WOA	ــــــــــــ	0.240	0.183	0.201
		MRAC-PI	7.149	0.133	0.101	0.150
Case 7: Random changes in wind speed		PI-PSO	6.744	0.220	0.229	0.221
		PI-WOA	6.621	0.217	0.184	0.152
		MRAC-PI	5.860	0.121	0.130	0.110
Case 8: Different disturbances in the system		PI-PSO	ــــــــــــ	0.263	0.647	0.381
		PI-WOA	ــــــــــــ	0.251	0.425	0.284
		MRAC-PI	7.175	0.153	0.293	0.160

## CASE 2—Sudden changes in wind speed

**Fig. 30:**
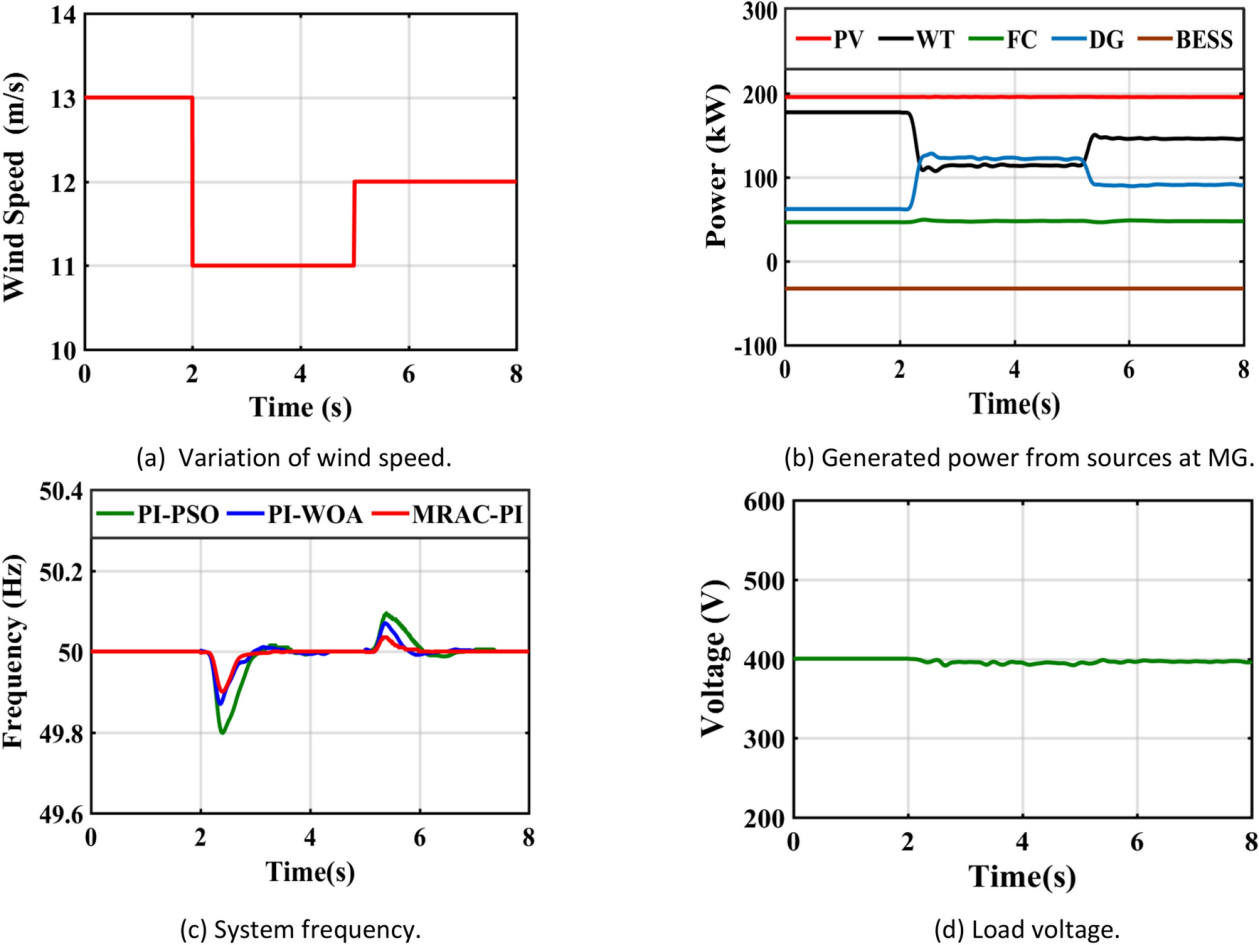
System response under case 2.

Figure 30(a) illustrates the step change in wind speed. Fluctuations in wind speed are crucial for assessing the performance of controllers in AC MGs, especially those that utilize RES like WT. These variations can significantly impact power generation and cause frequency imbalances. By modeling these changes, we can evaluate the controller’s capacity to adapt and keep the frequency within acceptable boundaries, thereby ensuring system stability. This assessment also facilitates the measurement of important performance indicators, such as Ts, ITAE, %Mp, and %Mus, which are vital for gauging the controller’s responsiveness and robustness. The MRAC-PI controller is particularly important for effective frequency regulation under these conditions. Compared to PI-PSO and PI-WOA controllers, the MRAC-PI controller shows enhanced accuracy in reacting to abrupt changes in wind speed, especially during periods of high efficiency and rapid weather changes. Table 8 and Fig. 30(b) depict the frequency response of the MG after a step change. Figure 30(c) also presents the output power from sources at MG. In this case, the wind speed drops at the 2-s point from 13 m/s to 11 m/s and then increases at the 5-s mark from 11 m/s to 12 m/s. At the 2-s point, the maximum power point tracking (MPPT) controller and boost converter match the WT’s rotor speed with the maximum power point (MPP) to maintain optimal power generation. This enhances DG’s power generation and compensates for the WT’s decreased power. The power of the DG rises from 63 kW to around 122 kW, whereas the power of the WT initially falls from 178 kW to roughly 114 kW, while the FC and PV keep on producing power at their highest levels. On the other hand, the WT’s power increases from 114 kW to roughly 147 kW at the 5-s point, while the DG’s power decreases from 122 kW to roughly 91 kW, while the FC and PV continue to produce power at their maximum rates. The MRAC-PI controller successfully maintains the stability of MG frequency despite these variations in wind speed. Lastly, Fig. 30(d) offers further insights by displaying the voltage levels within the system.

## CASE 3- Suddenly change in load

**Fig. 31:**
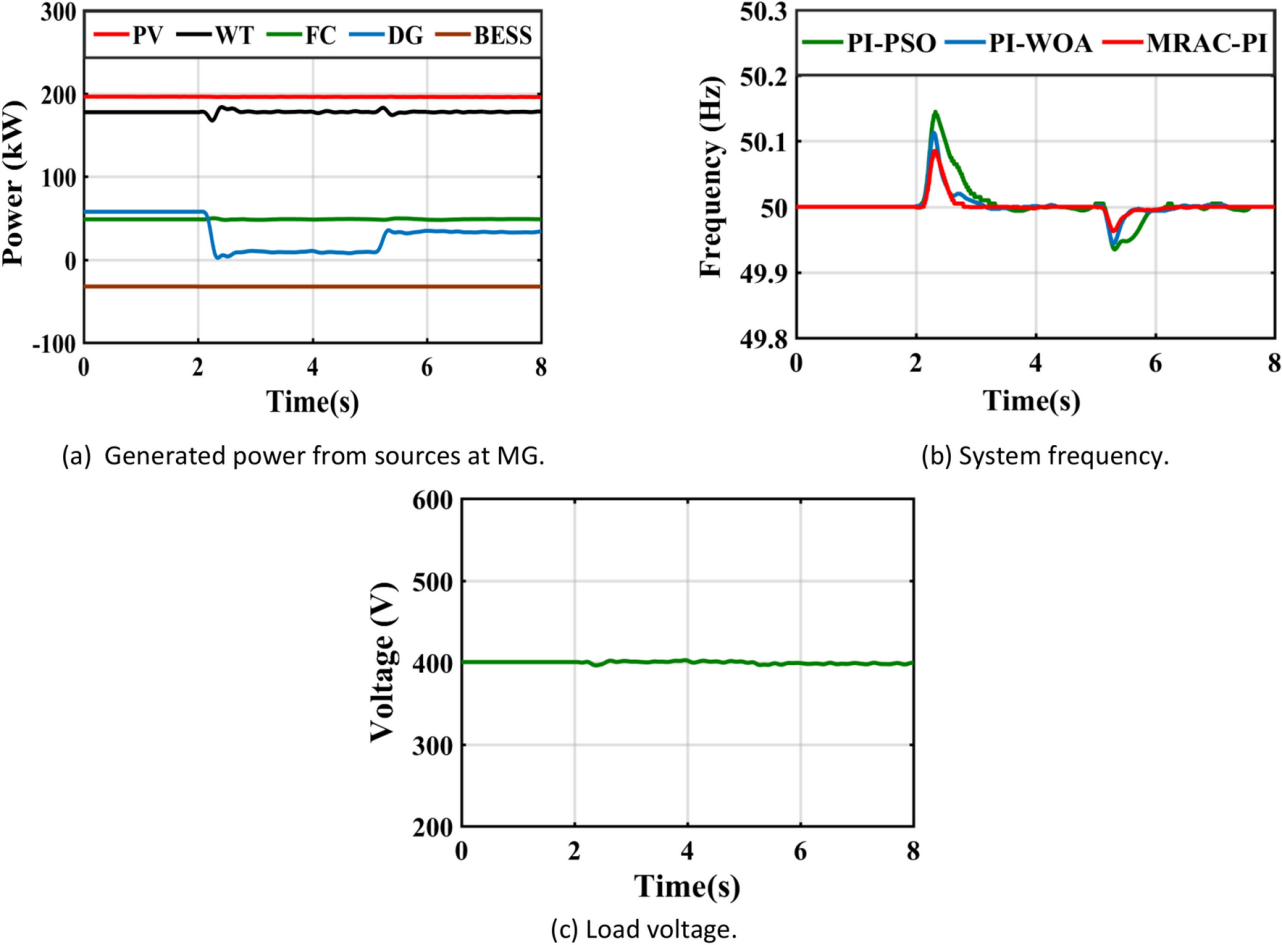
System response under case 3.

To assess how well the suggested control system performs in stabilizing AC bus voltage and attaining active power balance amongst MG components, it is imperative to consider the relevance of the sudden shift in load disturbance cases. At the 2- second point in this scenario, the MG’s overall power usage drops by 11.1%, from 450 to 400 kW, as shown in Fig. 31(a). However, the overall load demand rises to 425 kW from 400 kW at the 5-s point, a 6.25% increase. At the 2-s point, the DG power drops from 63 kW to roughly 9 kW, while the FC, PV, and WT all keep producing power at their highest levels. Nevertheless, the total load demand for MG increases by 6.25% at the 5-s point, going from 400 to 425 kW. As a result, during load fluctuations, the DG’s power output rises from 9 kW to around 34 kW; this means that the FC, PV, and WT are still producing power at their highest levels. Figure 31(b) and Table 8 provide a comparative analysis of the MRAC-PI, PI-PSO, and PI-WOA controllers in managing sudden load fluctuations, highlighting the superior performance of the MRAC-PI controller in maintaining optimal frequency control under these conditions. Additionally, Fig. 31(c) highlights the controller’s responsibility to keep the AC voltage within reasonable bounds, protecting the system’s power supply quality. This process increases the MG’s dependability during load shifts and emphasizes how vital efficient control systems are in overcoming the difficulties associated with integrating different energy sources.

## CASE 4 – Ramp changes in solar radiation

**Fig. 32:**
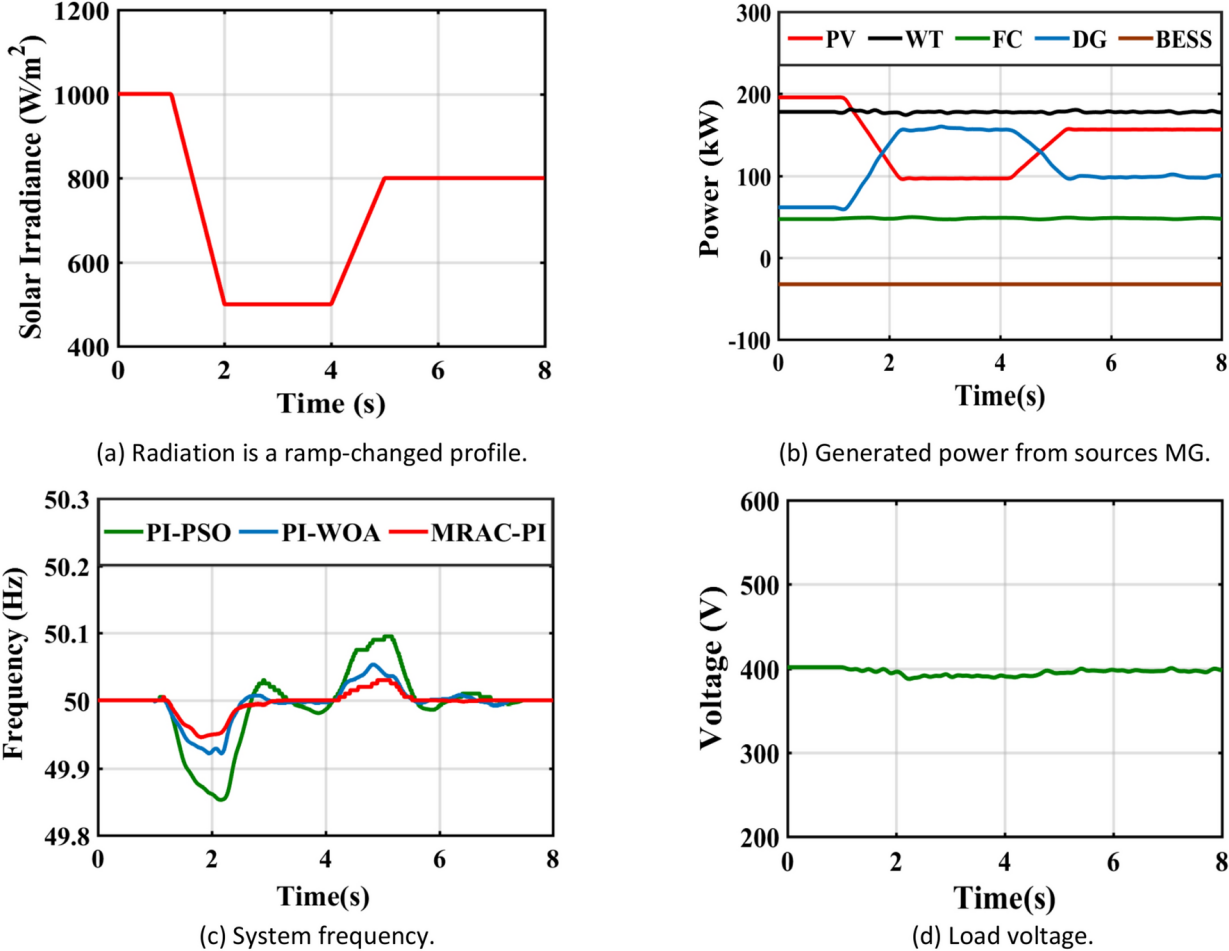
System response under case 4.

Figure 32(a) displays the ramp profile of solar radiation intensity over a designated timeframe. In such circumstances, the MRAC-PI controller is essential for achieving optimal frequency regulation. Compared to conventional PI-PSO and PI-WOA controllers, the MRAC-PI controller shows a markedly superior response and accuracy in adapting to changes in solar radiation intensity. Figure 32(b) shows the power output from sources at MG. As solar radiation diminishes, the power generated by the DEG increases correspondingly. Conversely, when solar irradiance rises, the output from the DG also escalates to meet the evolving energy demands. To assess system performance, a comparison is conducted among the MRAC-PI, PI-PSO, and PI-WOA controllers, focusing on critical performance indicators such as Ts, ITAE, %Mp, and %Mus. Table 8 and Fig. 32(c) provide a detailed analysis of the MG frequency response during ramp changes in solar radiation intensity. The MRAC-PI controller effectively stabilizes the MG frequency despite variations in solar intensity, demonstrating its ability to maintain consistent performance under differing conditions. Additionally, Fig. 32(d) offers further insights by illustrating the system’s voltage levels, contributing to a comprehensive understanding of the microgrid’s performance.

## CASE 5 – Ramp changes in wind speed

**Fig. 33:**
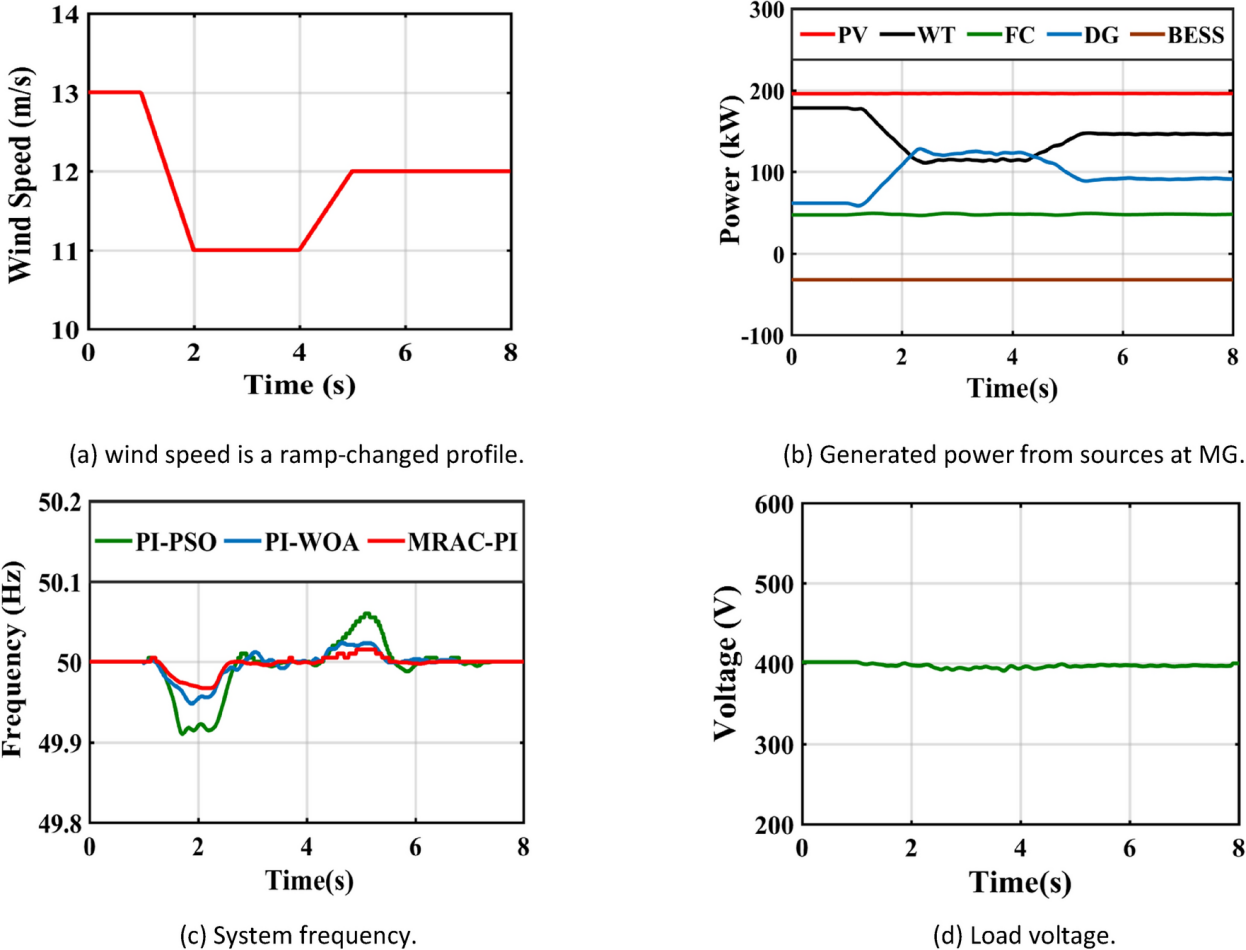
System response under case 5.

Figure 33(a) illustrates the ramp profile of wind speed over a specified period. In this scenario, the MRAC-PI controller ensures optimal frequency control. In contrast to PI-PSO and PI-WOA controllers, the MRAC-PI controller exhibits significantly enhanced responsiveness and precision in adjusting to fluctuations in wind speed. Figure 33(b) presents the power output from sources at MG. As wind speed decreases, the power produced by the DG correspondingly increases. Conversely, when wind speed increases, the output from the DG also rises to accommodate the changing energy requirements. A comparison is made among the MRAC-PI, PI-PSO, and PI-WOA controllers to evaluate system performance, emphasizing key performance metrics such as Ts, ITAE, %Mp, and %Mus. Table 8 and Fig. 33(c) offer a comprehensive analysis of the MG frequency response during ramp changes in wind speed. The MRAC-PI controller effectively stabilizes the MG frequency, even with fluctuations in wind speed, showcasing its capability to deliver consistent performance under varying conditions. Furthermore, Fig. 33(d) provides additional insights by depicting the voltage levels within the system, enhancing the overall understanding of the microgrid’s functionality.

## CASE 6 – Random changes in solar radiation

**Fig. 34:**
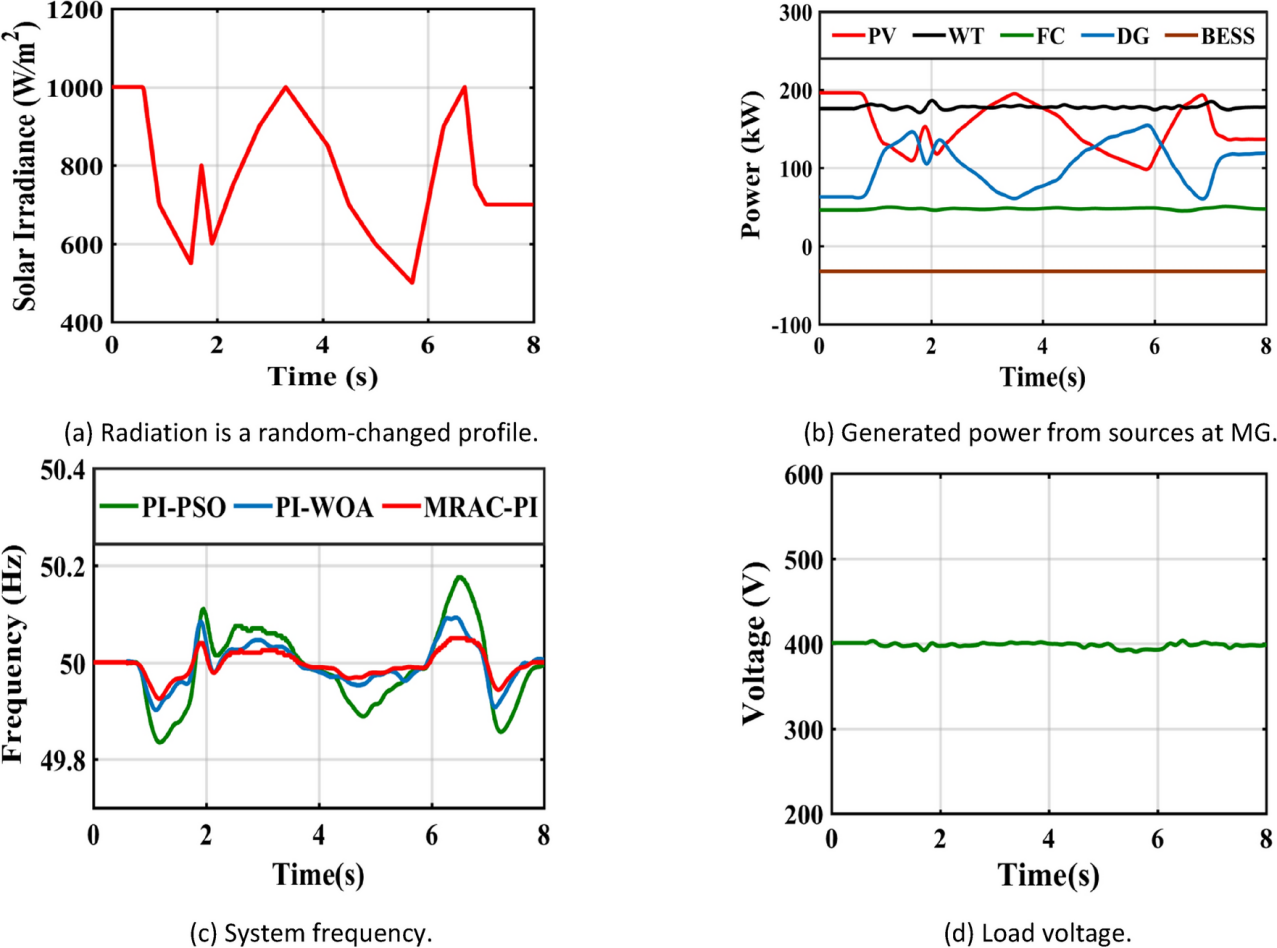
System response under case 6.

Figure 34(a) shows how solar radiation intensity changes over time. The MRAC-PI controller is crucial for optimal frequency regulation during these changes. It outperforms conventional PI-PSO and PI-WOA controllers in adapting to solar radiation variations. Figure 34(b) shows power output from sources at MG. As solar radiation decreases, DG power increases, and vice versa. Table 8 and Fig. 34(c) compare the performance of MRAC-PI, PI-PSO, and PI-WOA controllers using metrics like Ts, ITAE, %Mp, and %Mus. MRAC-PI stabilizes MG frequency during solar radiation changes, demonstrating consistent performance. Figure 34(d) shows system voltage levels, providing a complete picture of MG performance.

## CASE 7—Random changes in wind speed

**Fig. 35:**
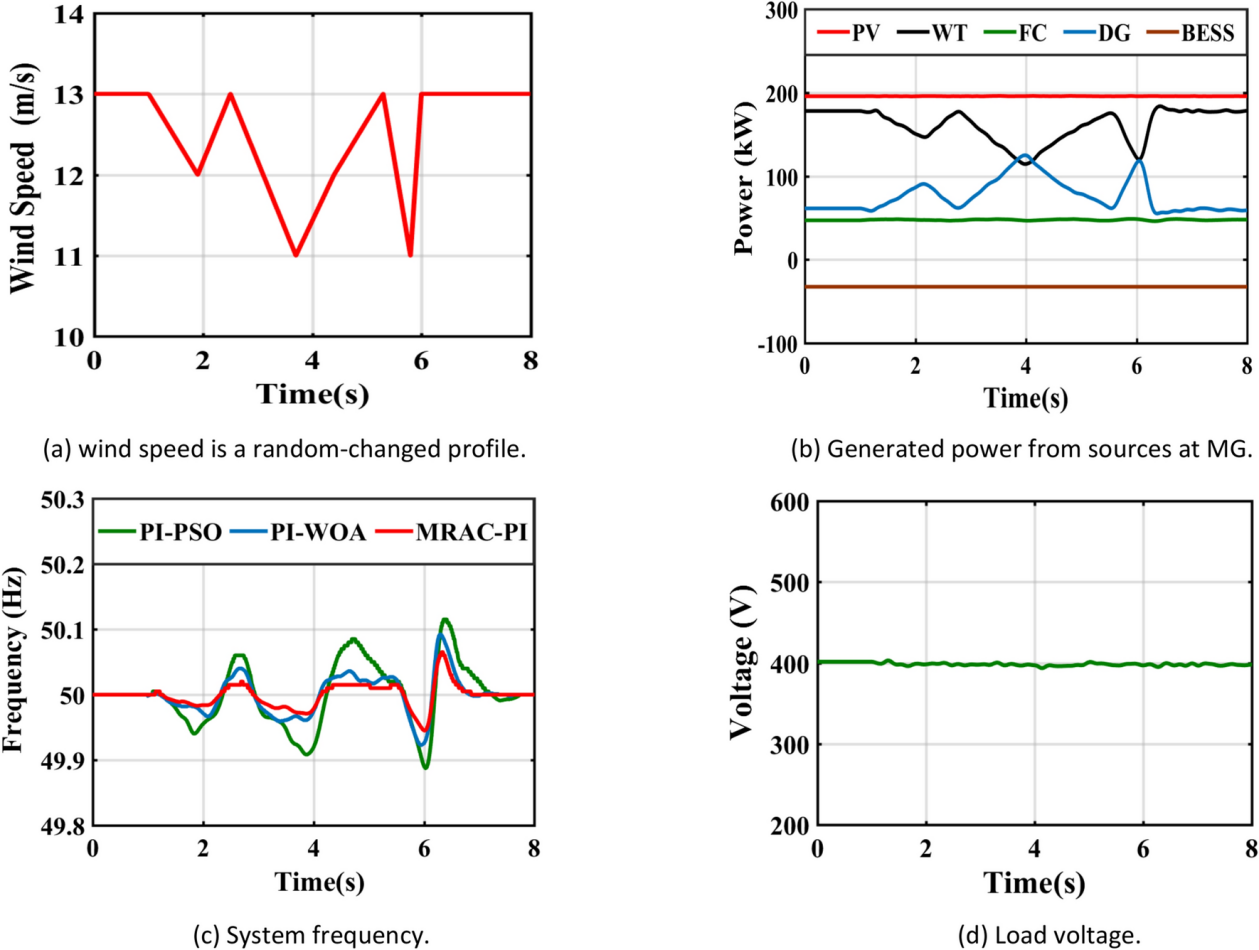
System response under case 7.

Figure 35(a) displays the random profile of wind speed over time, highlighting the MRAC-PI controller’s essential role in maintaining frequency control. Unlike PI-PSO and PI-WOA controllers, the MRAC-PI controller shows improved responsiveness and accuracy in adapting to wind speed changes. Figure 35(b) illustrates the power output from sources at MG, as wind speed drops, DG output increases, and vice versa. To assess system performance, comparisons are made among the MRAC-PI, PI-PSO, and PI-WOA controllers based on key metrics like Ts, ITAE, %Mp, and %Mus. Table 8 and Fig. 35(c) analyze the MG frequency response to wind speed variations. The MRAC-PI controller stabilizes frequency effectively despite wind fluctuations, demonstrating consistent performance. Additionally, Fig. 35(d) shows the system’s voltage levels, providing further insights into MG functionality.

## CASE 8 – Different disturbances in the system

**Fig. 36:**
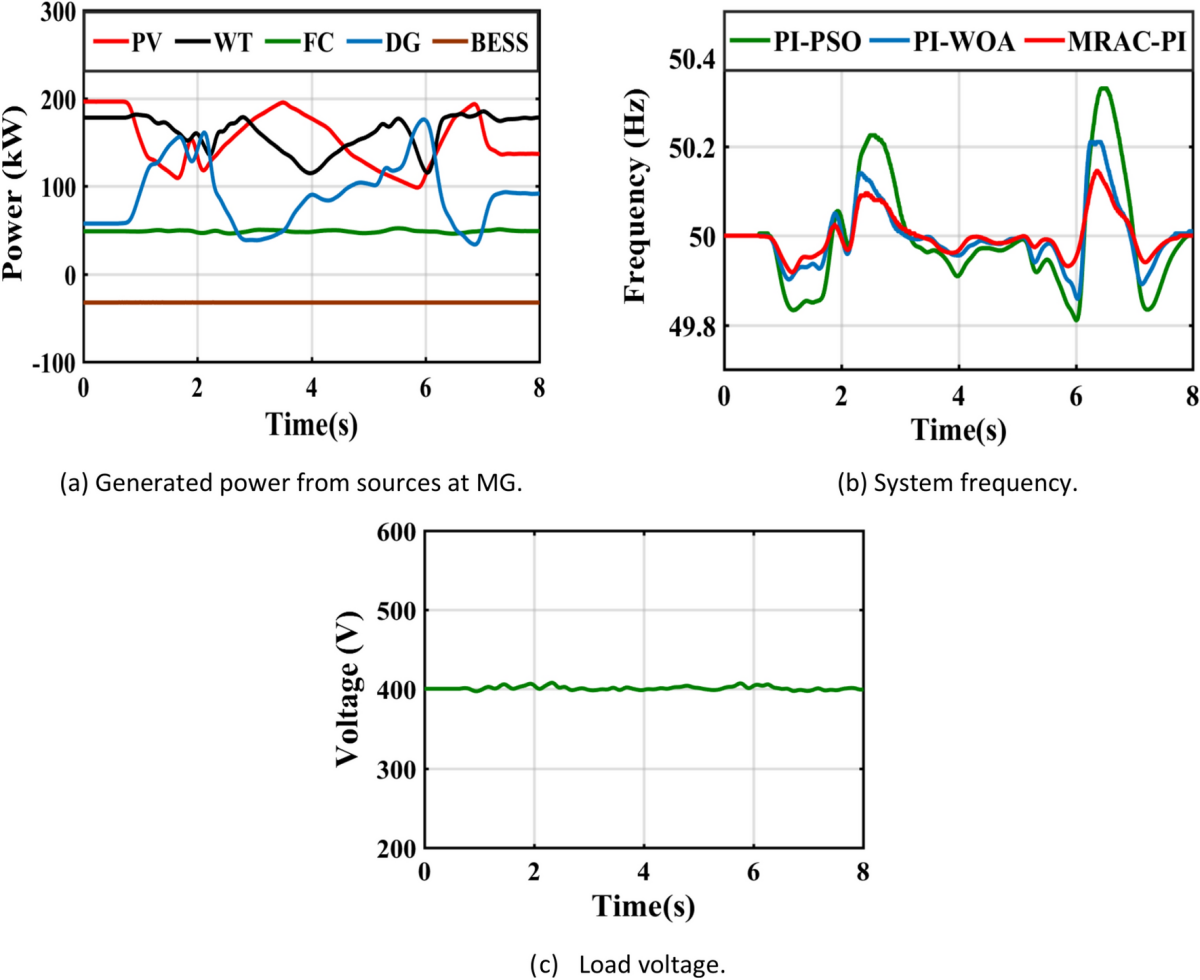
System response under case 8.

In this scenario, cases (3) sudden changes in load, (6) random changes in solar radiation, and (7) random changes in wind speed are interconnected and occur simultaneously. This approach ensures that the study addresses critical dynamic interactions between components, particularly the coupling of load fluctuations and renewable energy generation, thereby enhancing the technical accuracy of the work. Figure 36(a) visually depicts the power output from sources at MG. Table 8 and Fig. 36(b) comprehensively overview the MG frequency response. When comparing the performance of the MRAC-PI controller to PI-PSO and PI-WOA controllers, the MRAC-PI demonstrates a superior level of precision in addressing the conditions outlined in case 8. Finally, Fig. 36(c) offers additional insights by illustrating the voltage levels present in the system.

## Conclusion

This paper provides optimal design and techno-economic analysis of an islanded AC microgrid to cover the load of an international school in the New Administrative Capital, New Cairo, Egypt. The site was chosen because it serves as an example of new urban projects that emphasize sustainable energy sources, making it pertinent for settings of a similar nature worldwide. The islanded AC MG components were initially optimized for size and economic viability using HOMER. The simulation studies focused on the proposed area and utilized accurate load profile data and weather information. Various costs, like operating and maintenance (O&M), replacement, and capital, were considered, along with the operating life and efficiency of each HRES element. Other input parameters included the interest rate, evaluation of meteorological data, and project lifespan. To ensure the HRESs met the electricity demand of the residential area, optimization constraints such as operational savings, permitted capacity shortfall, load demand, and availability of resources were considered. Also, the system’s dynamic performance was investigated under different scenarios.

According to the HOMER simulation results, an islanded AC MG design with PV/WT/DG/FC/BESS/ELZ/HT is the most feasible option for the specified area. A 200 kW PV system, a 180-kW WT, a 141-kW converter, a 50.0 kW FC, a 50.0 kW ELZ, a 50.0 kg HT, a 180 kW DG, and a 686-kWh lead-acid battery are the optimal capacity of the MG sources. The outcomes show how each energy source contributes to the power demand, resulting in a TNPC of $1,775,300.00 and an LCOE of $0.153/kWh. By comparing our research with other studies, it becomes evident that it outperforms other studies regarding the LCOE.

The optimal setup of the MG obtained by HOMER was modeled in MATLAB/SIMULINK to study its dynamic performance under different scenarios. A model reference adaptive control—Proportional-Integral (MRAC-PI) controller is introduced to improve the dynamic performance and effectively regulate the system’s frequency and voltage. The performance of the MRAC-PI controller is assessed by contrasting it with the PI-PSO and PI-WOA controllers. Different disturbances, such as variations in wind speed, load demand, and solar irradiance, were introduced to evaluate the proposed control strategy for the MG alongside the MRAC-PI controller. The assessment relies on several performance indicators, including ITAE, Ts, %Mp, and %Mus of the system frequency. The findings indicate that the MRAC-PI controller significantly outperforms the PI-PSO controller across various scenarios. In Case 1, as solar radiation shifts from 1000 (W/m^2^) to 550 (W/m^2^), the MRAC-PI controller exhibits exceptional responsiveness, achieving an 86.11% reduction in overshoot, a 40.45% cut in undershoot, a 41.85% decrease in settling time, and a 44.02% reduction in ITAE. When solar radiation increases from 550 (W/m^2^) to 800 (W/m^2^), MRAC-PI continues to excel, reducing overshoot by 47.38%, undershoot by 88.71%, settling time by 28.34%, and ITAE by 45.81%. In Case 2, when wind speed changes from 13 to 11 m/s, MRAC-PI again showcases its remarkable agility, cutting overshoot by 100%, undershoot by 51.73%, settling time by 34.37%, and ITAE by 42.61%. As wind speed rises from 11 to 12 m/s, MRAC-PI maintains its strong performance, reducing overshoot by 63.55%, undershoot by 96%, settling time by 46.72%, and ITAE by 45.55%. In Case 3, with an 11.1% drop in total demand, MRAC-PI shows its responsiveness, achieving a 42.57% reduction in overshoot, a 58.33% decrease in undershoot, a 28.66% cut in settling time, and a 39.78% reduction in ITAE. When the load increases by 6.25%, MRAC-PI continues to perform well, reducing overshoot by 100%, undershoot by 43.08%, settling time by 36.86%, and ITAE by 39.78%. During ramp changes in solar radiation in Case 4, the MRAC-PI controller effectively meets the challenge, achieving a 68.42% reduction in overshoot, a 62.11% reduction in undershoot, a 14.83% decrease in settling time, and a 44.49% reduction in ITAE. In Case 5, when faced with ramp changes in wind speed, the MRAC-PI controller demonstrates its durability, reducing overshoot by 74.17%, undershoot by 63.54%, settling time by 18.52%, and ITAE by 45.33%. The effectiveness of MRAC-PI continues in Case 6, where random fluctuations in solar radiation result in a 70.38% reduction in overshoot, a 54.55% reduction in undershoot, a complete reduction in settling time (100%), and a 45.71% decrease in ITAE. In Case 7, with random variations in wind speed, the MRAC-PI controller delivers outstanding results, reducing overshoot by 43.23%, undershoot by 50.23%, settling time by 13.11%, and ITAE by 45%. Finally, in Case 8, where the system experiences various disturbances, the MRAC-PI controller once again showcases its effectiveness, achieving reductions in overshoot by 54.71%, undershoot by 58.01%, settling time by 100%, and ITAE by 41.83%. In summary, these results underscore the exceptional capabilities of the MRAC-PI controller in reducing Ts, ITAE, %Mp, and %Mus across various situations, showcasing its effectiveness in improving dynamic performance within control systems. Additionally, the findings reveal that the MRAC-PI controller swiftly reinstates the necessary frequency and upholds stability, even amid multiple disturbances. In contrast, the PI-PSO and PI-WOA controllers demonstrate insufficient performance in all tested scenarios.

Future work could enhance this research by integrating reliability metrics such as Loss of Power Supply Probability (LPSP) and Energy Not Supplied (ENS) into our analysis. These quantitative measures will enable a more comprehensive evaluation of the system’s robustness and reliability, thereby enhancing the overall assessment of the proposed energy configuration. We could also explore thermal management strategies for critical components, including fuel cells and batteries, to enhance overall system efficiency. This work can also be extended by designing optimal MGs in other regions with various characteristics.

## Supplementary Information

Supplementary Information.

## Data Availability

The data supporting this study’s findings are available to the corresponding author upon request.
