# Supplementary material for: A case study of optimal design and techno-economic analysis of an islanded AC microgrid
Source: Sci Rep. 2025 Apr 11;15:12397. doi: 10.1038/s41598-025-94506-z (PMC11992058; doi:10.1038/s41598-025-94506-z)
Supplement: Supplementary file 1 — Supplementary Information. [file 41598_2025_94506_MOESM1_ESM.docx]

**Appendix A.** An electrical overview of the data output and input for every component in the optimized power system.

| Component | data | Value | data | Value |
| --- | --- | --- | --- | --- |
| PV | Minimum Output | 0 kW | Maximum Output | 186 kW |
|  | PV Penetration | 46.7 % | Hours of Operation | 4,385 hrs/yr |
|  | Levelized Cost | 0.0468 $/kWh | Rated Capacity | 200 kW |
|  | Mean Output | 36.8 kW | Mean Output | 884 kWh/d |
|  | Capacity Factor | 18.4 % | Total Production | 322,526 kWh/yr |
| WT  Eocycle EO20 | Minimal Output | 0 kW | Maximum Generation | 182 kW |
|  | Wind Penetration | 118% | Operating Hours | 7,622 hrs/yr |
|  | Levelized Cost | 0.0238 $/kWh | Total Rated Capacity | 180 kW |
|  | Mean Output | 92.7 kW | Capacity Factor | 51.5 % |
|  | Total Production | 812,486 kWh/yr |  |  |
| Fuel Cell | Electrical Production | 22,439 kWh/yr | Average Power Generation | 35.9 kW |
|  | Minimum Power Generation | 0.407 kW | Maximum Power Generation | 50.0 kW |
|  | Fuel Consumption | 1,346 kg | Specific Fuel Consumption | 0.0600 kg/kWh |
|  | Fuel Energy Input | 44,877 kWh/yr | Mean Electrical Efficiency | 50.0 % |
|  | Operating Hours | 625 hrs/yr | Number of Starts | 276 stars/yr |
|  | Life of Operations | 64.0 yr | Capacity Factor | 5.12 % |
|  | Fixed Production Expense | 8.75 $/hr | Marginal Generation Cost | 0 $/kWh |
| Diesel Generator | Electrical Production | 28,921 kWh/yr | Average Power Generation | 52.8 kW |
|  | Minimum Electrical Output | 45.0 kW | Maximum Power Generation | 108 kW |
|  | Fuel Consumption | 9,543 L | Specific Fuel Consumption | 0.330 L/kWh |
|  | Fuel Energy Input | 93,906 kWh/yr | Mean Electrical Efficiency | 30.8 % |
|  | Hours of Operation | 548 hrs/yr | Number of Starts | 146 stars/yr |
|  | Operational Life | 27.4 yr | Capacity Factor | 1.83 % |
|  | Fixed Generation Cost | 16.4 $/hr | Marginal Generation Cost | 0.236 $/kWh |
| Battery: Hoppecke 24 OPzS 3000 | Batteries | 96.0 qty | String Size | 24.0 batteries |
|  | Strings in Parallel | 4.00 strings | Bus Voltage | 48.0 V |
|  | Average Energy Cost | 0 $/kWh | Energy In | 102,937 kWh/yr |
|  | Energy Out | 88,551 kWh/yr | Storage Depletion | 27.4 kWh/yr |
|  | Losses | 14,413 kWh/yr | Annual Throughput | 95,487 kWh/yr |
|  | Autonomy | 6.10 hr | Storage Wear Cost | 0.0709 $/kWh |
|  | Nominal Capacity | 686 kWh | Usable Rated Capacity | 480 kWh |
|  | Lifetime Throughput | 971,357 kWh | Expected Life | 10.2 yr |
| Electrolyzer | Mean Output | 0.157 kg/hr | Minimum Output | 0 kg/hr |
|  | Maximum Output | 1.08 kg/hr | Total Production | 1,380 kg/yr |
|  | Specific Consumption | 46.4 kWh/kg | Rated Capacity | 50.0 kW |
|  | Mean Input | 7.31 kW | Minimum Input | 0 kW |
|  | Maximum Input | 50.0 kW | Total Input Energy | 64,025 kWh/yr |
|  | Capacity Factor | 14.6 % | Hours of Operation | 1,605 hr/yr |
| Hydrogen Tank | Hydrogen Storage Capacity | 50.0 kg | Energy Storage Capacity | 1,667 kWh |
|  | Tank Autonomy | 21.2 hr | Content at the Beginning of the Year | 5.00 kg |
|  | Content at the End of the Year | 38.4 kg |  |  |
| Converter: Studer AJ 2400-24 | Hours of Operation | 3,827 hrs/yr | Energy Out | 181,873 kWh/yr |
|  | Energy In | 193,482 kWh/yr | Losses | 11,609 kWh/yr |
|  | Capacity | 141 kW | Mean Output | 20.8 kW |
|  | Minimum Output | 0 kW | Maximum Output | 128 kW |
|  | Capacity Factor | 14.7 % |  |  |
